# The suppressive role of GLS in radiosensitivity and irradiation-induced immune response in LUAD: integrating bioinformatics and experimental insights

**DOI:** 10.3389/fimmu.2025.1582587

**Published:** 2025-04-16

**Authors:** Peicheng Jiang, Zhifeng Jiang, Su Li, Ye-Xiong Li, Yuqiong Chen, Xinyan Li

**Affiliations:** ^1^ State Key Laboratory of Molecular Oncology and Department of Radiation Oncology, National Cancer Center/National Clinical Research Center for Cancer/Cancer Hospital, Chinese Academy of Medical Sciences and Peking Union Medical College, Beijing, China; ^2^ Department of Liver Surgery & Transplantation, Liver Cancer Institute, Zhongshan Hospital, Fudan University, Key Laboratory of Carcinogenesis and Cancer Invasion, Ministry of Education, Shanghai, China; ^3^ Department of Cardiology, Zhongshan Hospital, Fudan University, Shanghai Institute of Cardiovascular Diseases, Shanghai, China; ^4^ Department of Cardiology, National Clinical Research Center for Interventional Medicine, Shanghai, China; ^5^ Department of Cardiology, The Affiliated Suzhou Hospital of Nanjing Medical University, Suzhou Municipal Hospital, Gusu School, Nanjing Medical University, Suzhou, China

**Keywords:** glutamine metabolism, lung cancer, immunity, tumor microenvironment, prognostic model

## Abstract

**Background:**

Radiotherapy elicits immune activation, thereby synergistically enhancing systemic tumor control when combined with immunotherapy. Glutaminase (GLS), a key enzyme for glutamine metabolism, has been found to regulate glutamine availability within tumor microenvironment (TME). However, the precise mechanisms through which GLS modulates radiosensitivity and irradiation-induced immune responses in lung adenocarcinoma (LUAD) and its clinical value remain to be fully elucidated.

**Methods:**

We employed bulk RNA-seq and single-cell transcriptomics to explore the role of GLS expression in radiosensitivity and immune infiltration. The bioinformatic results were validated by *in vitro* and *in vivo* experiments. Co-culture assays and flow cytometry were used to validate the impact of GLS expression on CD8^+^ T cell activation and cytotoxicity. Moreover, a GLS-DSBr (double strand break repair) prognostic model was developed using machine learning with data from 2,066 LUAD patients.

**Results:**

*In vitro* and *in vivo* experiments demonstrated that GLS silence inhibited DSB repair and promoted ferroptosis, therefore enhancing radiosensitivity. Single-cell and spatial transcriptomics revealed the immunomodulatory effects of GLS expression in the TME. Further, Co-culture assays and flow cytometry experiments indicated that silencing GLS in LUAD cells potentiated the activation and cytotoxicity of CD8^+^ T cells in the context of radiotherapy. The GLS-DSBr model demonstrated robust predictive performance for overall survival, as well as the efficacy of radiotherapy and immunotherapy in LUAD. The applicability of GLS-DSBr model was further validated through pan-cancer analysis.

**Conclusion:**

In the contexts of radiotherapy, GLS downregulation exerts dual regulatory effects by modulating ferroptosis and remodeling the immune landscapes, particularly enhancing CD8^+^ T cell cytotoxicity. Our work suggests that strategies preferentially targeting GLS in tumor cells may represent promising and translatable therapeutic approaches to promote antitumor efficacy of radiotherapy plus immune checkpoint blockade in LUAD patients. Furthermore, the established GLS-DSBr model serves as a robust predictive tool for prognosis and effects of radiotherapy and immunotherapy, which assists personalized treatment optimization in LUAD.

## 1 Introduction

Lung cancer represents the most prevalent and lethal malignancy in China, with lung adenocarcinoma (LUAD) constituting approximately 40% of all cases (1). Radiotherapy, a cornerstone in lung cancer treatment, exerts dual therapeutic effects through direct tumor cell eradication and systemic immune activation (2, 3). However, the clinical efficacy of radiotherapy is frequently compromised by intrinsic radioresistance, which may result in treatment failure and subsequent tumor recurrence or metastasis (2, 4). This resistance primarily stems from enhanced DNA double-strand break (DSB) repair capacity, as DSBs represent the predominant form of radiation-induced DNA damage that ultimately leads to cancer cell death (5). Consequently, elucidating the molecular mechanisms governing radiosensitivity has become paramount for optimizing therapeutic outcomes.

The advent of immunotherapy has fundamentally transformed the clinical paradigm for advanced non-small cell lung cancer (NSCLC) treatment. In this new era, the strategic combination of immune checkpoint inhibitors (ICIs) with other therapeutic modalities, particularly radiotherapy, has emerged as a promising approach to enhance systemic antitumor immunity (6). This combination strategy, termed immunoradiotherapy (iRT), capitalizes on radiotherapy’s unique ability to convert immunologically “cold” tumors into “hot” tumors, thereby potentially augmenting ICI efficacy (7–9). While preclinical studies have consistently demonstrated the immune-modulatory effects of radiotherapy within the tumor microenvironment (TME), translating these findings into consistent clinical benefits remains challenging. The PACIFIC trial established durvalumab as a standard consolidation therapy following concurrent chemoradiotherapy (10, 11), yet the subsequent PACIFIC-2 trial failed to demonstrate significant survival benefits with this combination approach (12). These discordant outcomes underscore the complex interplay between radiotherapy and immunotherapy and highlight the critical need to identify novel therapeutic targets that can enhance radiotherapy-induced immune activation while overcoming immunosuppressive TME barriers. Addressing these challenges represents a crucial frontier in optimizing iRT efficacy and improving patient outcomes.

As the most abundant circulating amino acid, glutamine serves as a crucial nutrient source for cancer cells, supporting fundamental cellular processes including DNA repair and redox homeostasis (13, 14). Notably, recent studies have demonstrated that enhanced glutamine metabolism not only promotes radioresistance through maintaining cellular redox balance and facilitating DNA damage repair but also contributes to immunosuppression within the TME (15–17). These findings suggest that targeting glutamine metabolic pathways may represent a promising therapeutic strategy to enhance radiotherapy efficacy. Among the key regulators of glutamine metabolism, glutaminase (GLS) has garnered particular attention due to its pivotal role in catalyzing the conversion of glutamine to glutamate, the rate-limiting step in glutamine catabolism (18). Although several studies have explored the metabolic roles of GLS in cancer progression and therapy (18, 19), the precise mechanisms through which GLS modulates radiosensitivity in LUAD remains poorly understood. Moreover, compelling evidence suggests that tumor cells exploit their metabolic advantage to deplete extracellular glutamine, creating a nutrient-deprived TME that compromises CD8^+^ T cell function and antitumor immunity (20–23). This metabolic competition raises intriguing questions about whether and how GLS-mediated glutamine metabolism influences radiotherapy-induced immune responses. Therefore, the complex interplay between GLS and immune modulation in the contexts of radiotherapy warrants thorough investigation.

Ferroptosis, an iron-dependent form of regulated cell death driven by lipid peroxidation, has emerged as a promising avenue to enhance radiotherapy efficacy through synergistic effects with radiation-induced DNA damage (24–28). Emerging evidence suggests that the glutamine metabolism exerts a critical influence on ferroptosis by modulating cellular redox homeostasis, which provides mechanistic insight for developing therapeutic strategies targeting the ferroptosis-glutamine metabolism axis (29, 30). As a key enzyme in glutamine catabolism, GLS may modulate ferroptosis sensitivity, potentially influencing radiotherapy outcomes in LUAD. However, the specific role of GLS in regulating radiation-induced ferroptosis and its therapeutic implications remain unexplored, warranting further investigation to develop novel strategies for overcoming radioresistance.

This study comprehensively investigates the role of GLS in regulating radiosensitivity and immune modulation in LUAD. Through a multi-dimensional approach, we demonstrate that GLS inhibition enhances radiosensitivity by promoting ferroptosis and reprogramming the TME to potentiate anti-tumor immunity. We further establish the GLS-DSBr (DSB repair) model as a robust predictive tool for patient prognosis and treatment response, particularly in predicting the efficacy of radiotherapy and immunotherapy, validated across LUAD cohorts and pan-cancer analyses. Notably, the pan-cancer analysis revealed significant variations in the TME between high- and low-risk groups, demonstrating the model’s generalizability. These findings not only provide mechanistic insights into the metabolic regulation of radioresistance but also offer a translational framework for optimizing precision immunoradiotherapy. The potential integration of GLS inhibition with other immune checkpoint inhibitors, such as CTLA-4, may further enhance therapeutic efficacy, representing a promising direction for future combination strategies in LUAD treatment.

## 2 Materials and methods

The flow diagram for this study is presented in the *Graphical abstract* (**Supplementary Figure S1**).

### 2.1 Data collection and processing

Data from RNA sequencing and related clinical details were obtained from The Cancer Genome Atlas (TCGA) through the UCSC XENA portal (https://xena.ucsc.edu/). Transcriptomic and clinical data for the datasets GSE31210, GSE72094, GSE50081, GSE37745, GSE68456, and GSE131907 were downloaded from the Gene Expression Omnibus (GEO) repository. Immunotherapy-related data were sourced from GSE135222, which includes LUAD patients undergoing anti-PD-1/PD-L1 therapy (31). The analysis did not include patients who had incomplete survival data. And a total of 2,066 eligible patients were retained. Single-cell RNA sequencing data, encompassing 208,395 cells derived from LUAD, were obtained from GSE131907 (32). Spatial transcriptomic data were sourced from GSE189487 (33). The gene set for 18 types of programmed cell death (PCD) was obtained from Hu et al. (34).

### 2.2 Quality control and annotation of single-cell data

For the single-cell dataset, cells were excluded from further analysis if their Unique Molecular Identifier (UMI) counts were either below 200 or exceeded 10,000, or if more than 10% of their RNA expression was derived from mitochondrial genes, indicating poor quality.

After quality control, logarithmic normalization was applied to the remaining cells, and the top 2,000 highly variable genes (HVGs) were selected using the Seurat package (v4.3.0.1). Principal component analysis (PCA) was performed on the HVGs, followed by the construction of a shared nearest neighbor (SNN) graph and unified manifold approximation and projection (UMAP) using the Louvain algorithm, with the first 30 principal components used for clustering. Major cell type annotations for the single-cell data were derived from Kim et al. (32). Further subpopulation classification of T cells was conducted based on established cell markers (35).

### 2.3 The advanced analysis of single-cell RNA sequencing

To assess the distribution of clusters, we computed the ratio of observed to expected cell counts (Ro/e) for each cell type cluster among various groups. A higher Ro/e value signifies a stronger enrichment (36). The AUCell package was used to compute the AUCell score for various signature genes in individual cells within the clusters (37). To mitigate the impact of data sparsity, we applied the MetaCell algorithm to group homogeneous cells into metacells (38), thereby representing the overall structure of the single-cell RNA sequencing (scRNA-seq) data. Cellchat was employed to deduce, display, and examine communication between cell clusters (39).

### 2.4 Functional analysis

An enrichment analysis was carried out using the outcomes of the differential expression analysis using the ‘limma’ package. Gene set enrichment analysis (GSEA) and gene set variation analysis (GSVA) were conducted using the ‘clusterProfiler’ package (40), with gene sets sourced from MsigDB (The Molecular Signatures Database) and GO (Gene Ontology). METAFlux, an algorithm based on Metabolic Flux Balance Analysis (FBA), characterizes metabolic pathways in a nutrient-sensing context and predicts metabolic fluxes (41). We employed METAFlux to assess the glutamine and glutamate metabolic flu. To assess immune cell infiltration, multiple algorithms were employed, including TIMER 2.0 (42) and Cibersort (43). The ESTIMATE package was implemented to calculate the ESTIMATE score, tumor purity, and stromal score (44).

### 2.5 Spatial transcriptome RNA sequencing analysis

Spatial transcriptome data were normalized using SCTransform, and SpatialFeaturePlot was employed to visualize the spatial expression of genes. SpaCET was employed to assess the quantity of cancer cells, determine the stromal and immune cell lineage scores, and assess the cellular lineages and intercellular interactions within the TME (45).

### 2.6 Construction of prognostic signature by machine learning approaches.

First, we performed differential expression analysis on two groups with different GLS expression levels in the TCGA_LUAD dataset. The differentially expressed genes were intersected with the DNA damage gene set (GO: 0006302). Subsequently, univariate Cox analysis was conducted, and 76 genes associated with prognosis were identified. Using these genes, we employed 10 algorithms and trained 101 algorithmic combinations. These models were developed using the leave-one-out cross-validation (LOOCV) framework and trained on the TCGA-LUAD cohort. The models were then validated on four independent datasets (GSE31210, GSE72094, GSE50081, GSE37745). Harrell’s concordance index (C-index) was calculated across all datasets for each model, and the model with the top average C-index was selected as the optimal model.

### 2.7 Genomic analysis

Genomic Copy number variation (CNV) and Single nucleotide polymorphism (SNP) data were downloaded using the TCGAbiolinks package. CNV data were processed and analyzed using GISTIC2 (https://www.genepattern.org/), a tool designed for identifying and visualizing significant copy number alterations across samples. Visualization of the CNV results was performed using the maftools package.

### 2.8 Cell culture and treatments

Human lung cancer cell lines A549, NCI-H23, HCC827, and NCI-H226, as well as the mouse Lewis lung carcinoma (LLC) cell line, were sourced from the Cell Resource Center of the Institute of Basic Medical Sciences (CAMS, China). The human embryonic kidney 293T cell line was acquired from the American Type Culture Collection (ATCC). A549, NCI-H23, NCI-H226, and 293T cells were cultured in DMEM (GIBCO, USA), while HCC827 and LLC cells were grown in RPMI 1640 medium (GIBCO, USA). Cells were preserved in a humidified incubator at 37°C with a CO_2_ concentration of 5% (Thermo Scientific, USA). Irradiation was administered at the specified dose, and cells were cultured for the indicated time prior to subsequent experiments.

### 2.9 Lentiviral vectors and lentivirus packaging

Lentiviral vectors were constructed using the pLKO.1-PuroR backbone plasmid to generate GLS knockdown constructs (TsingKe Biotechnology, China). To produce lentiviruses, HEK293T cells were simultaneously transfected with shRNA plasmids, along with psPAX2 and pMD.2G packaging plasmids (Addgene, USA). Lentiviral supernatants were collected 48 hours post-transfection and used to infect A549 and NCI-H23 tumor cells. Infected cells were selected with puromycin (Solarbio, China) for 48 hours to establish stable transfectants.

### 2.10 Glutamine level detection

Intracellular glutamine levels in lung cancer cells were measured using a glutamine detection kit (BTK133, Bioswamp, China). Cell lysates or standards were mixed with the working solution and incubated at 37°C for 30 minutes. After measuring the optical density (OD) at 450 nm, the glutamine concentration in the samples was determined by referencing the OD values against the standard curve.

### 2.11 Glutamate level detection

Intracellular glutamate levels in cells were measured using a glutamate detection kit (BTK048, Bioswamp, China). The samples or standards were mixed with the working solution and incubated at 37°C for 20 minutes. The OD was recorded at 450 nm, and glutamate levels in the samples were calculated by matching the OD readings to the standard curve.

### 2.12 Western blot

Cells were lysed in NETN buffer, followed by centrifugation to collect the protein-containing supernatant. Protein samples were mixed with loading buffer, boiled, separated by electrophoresis, and transferred. The membranes were blocked and incubated with primary and secondary antibodies. The primary antibodies included anti-GLS (ab156876, Abcam), anti-γ-H2AX (05-636, Sigma), anti- Glutathione Peroxidase 4 (GPX4, ab125066, Abcam), and anti-β-actin (81115-1-RR, Proteintech).

### 2.13 Colony formation assay

GLS-depleted A549 and NCI-H23 cells, along with their corresponding NC cells, were seeded in 6-well plates and exposed to IR at the indicated doses (2, 4, 6, and 8 Gy) for 10 to 14 days. After fixing and staining, the colonies were counted and the numbers were normalized based on plating efficiencies.

### 2.14 Immunofluorescence staining

GLS-depleted A549 and NCI-H23 cells, along with NC cells, were seeded onto coverslips and incubated for 24 hours before being treated with IR (1 Gy). Following treatment (1 or 8 hours with or without IR), cells were fixed and permeabilized, followed by overnight incubation with primary antibodies against γ-H2AX (05-636, Sigma) at 4°C. Following washing, cells were treated with secondary antibodies for an hour at room temperature, and DAPI was used to counterstain the nuclei. After further washing with PBS, cells were mounted with anti-fade solution on glass slides and visualized using a fluorescence microscope. For foci counting, more than 100 cells per group were analyzed to standardize the data.

### 2.15 Coculture assay and flow cytometry

GLS-depleted A549 cells and corresponding NC cells were irradiated with 10 Gy and seeded into 24-well plates (8 × 10³ cells/well). Human peripheral blood mononuclear cells (PBMCs) were activated with anti-CD3 (0.5 μg/mL, 300302, Biolegend), anti-CD28 (2 μg/mL, 302902, Biolegend) antibodies, and human IL-2 (50 ng/mL, 42212, Peprotech), and then added to the A549 cells at an effector-to-target ratio of 25:1. After 24 hours, PBMCs were stained with CD45-FITC, CD3-PerCP, CD8-APC/Cy7, and CD69-APC antibodies (Biolegend). After 3 days, PBMCs were stained with CD45-FITC, CD3-PerCP, CD8-APC/Cy7, and GranzymeB-PE antibodies (Biolegend). For flow cytometric analysis, cell samples were subjected to surface marker staining by incubation with fluorophore-conjugated antibodies for 30 minutes at 4°C. Intracellular staining procedures involved the application of fixation and permeabilization buffers (Tonbo Biosciences). Isotype controls were conducted using matched nonspecific IgG. Following two washes, the samples were acquired on a flow cytometer (BD Biosciences). To quantify tumor cell apoptosis in the co-culture system, GLS-depleted A549 cells and corresponding NC cells were irradiated with 10 Gy and seeded into 96-well plates (4 × 10^4^ cells/well) after CFSE staining (1 μM, 565082, BD Biosciences). Activated human PBMCs were added at an effector-to-target ratio of 6:1. After 12 hours, adherent tumor cells and supernatant were collected, stained with Annexin V-APC (Biolegend) and Propidium Iodide (Solarbio), and analyzed by flow cytometry. The flow cytometry data were analyzed using FlowJo software version 10.

### 2.16 Cell counting kit-8 assay and lactate dehydrogenase release assays

Cell viability was assessed using Cell counting kit-8 (CCK-8, Dojindo, Shanghai, China). Cells were plated and subjected to irradiation at 10 Gy. At specified time intervals, Fresh medium with 10% CCK-8 reagent was used to replace the old medium, and the cells were incubated at 37°C under 5% CO_2_ for 1 hour. Subsequently, absorbance was measured at 450 nm. Lactate dehydrogenase (LDH) release was quantified with an LDH assay kit (Nanjing Jiancheng, China). Cells were seeded into 6-well plates and exposed to IR. After treatment, the culture supernatants were harvested, and cells were lysed with 1.5% Triton X-100. The lysates and supernatants were then reacted with coenzyme I and 2,4-dinitrophenylhydrazine for 15 minutes at 37°C. Absorbance readings were taken at 440 nm to determine LDH activity.

### 2.17 Lipid peroxidation measurements

To assess the extent of lipid peroxidation within cells, we measured the level of intracellular lipid peroxidase (LPO) activity using a commercial assay kit (Cayman, USA). Additionally, the concentration of malondialdehyde (MDA), a secondary product of lipid peroxidation, was quantified with an MDA assay kit (Nanjing Jiancheng, China). In parallel, reactive oxygen species (ROS) levels were evaluated as indicators of oxidative stress. Intracellular ROS was detected using two fluorescent probes, DCFH-DA and dihydroethidium (DHE; Beyotime, China).

### 2.18 GPX4 activity

To determine GPX4 enzymatic activity, cells were lysed and mixed with a buffer composed of Tris (pH 7.5), EDTA, NaN_3_, and Triton-X 100, ensuring the absence of oxidizing agents. The lysates were then homogenized and supplemented with glutathione reductase, NADPH, reduced glutathione (GSH), and H_2_O_2_. GPX4 activity was quantified by monitoring the decrease in NADPH absorbance at 340 nm.

### 2.19 Tumor xenograft

For the establishment of subcutaneous xenograft models, 5 × 10^6^ specified cells were resuspended in a 100 μL mixture of Matrigel (Corning, USA) and PBS and administered into the dorsal side of male BALB/c nude mice (Hua Fukang Biological Technologies, China). Once tumor volumes reached 60–120 mm³, mice were randomly allocated into irradiated and non-irradiated groups. In the IR group, localized tumors were subjected to daily irradiation at 2 Gy for one week. Tumor dimensions were assessed with calipers, and volume was calculated using the formula 0.5 × length × width². Following treatment, mice were euthanized at predetermined time points, and tumor tissues were promptly harvested for further Western blot and immunohistochemistry (IHC) analysis.

### 2.20 Hematoxylin & eosin and IHC staining

Tumor tissues were fixed, processed for paraffin embedding, and sectioned into 4 μm slices. Hematoxylin & Eosin (H&E) staining was conducted following standard protocols. For IHC, antigen retrieval was performed. Sections were subsequently incubated overnight at 4°C with primary antibodies against GPX4 (ab125066, Abcam), γ-H2AX (05-636, Sigma) and Ki-67 (28074-1-AP, Proteintech). On the following day, the sections were washed and incubated with horseradish peroxidase (HRP)-conjugated secondary antibodies. Nuclear counterstaining was performed using hematoxylin. The extent of IHC-positive expression was quantified as the proportion of the stained region compared to the entire tissue area, calculated as (stained area/total area) × 100%, using ImageJ software for analysis.

### 2.21 Ethics statement

All animal experiments were performed in accordance with relevant guidelines and regulations. The study protocol was approved by the Animal Ethics Committee of Cancer Hospital of Chinese Academy of Medical Sciences and Peking Union Medical College. The studies involving humans were approved by the Committee for the Ethics Review of Research Involving Human Subjects of the Cancer Hospital of the Chinese Academy of Medical Sciences. All experiments were conducted according to the Declaration of Helsinki principles.

### 2.22 Statistical analysis

The statistical analyses were conducted using two-tailed unpaired t-tests for comparisons between two groups, and one-way ANOVA or two-way ANOVA for multiple comparisons, as appropriate. R software was used to conduct all analyses (version 4.2.1). Pearson correlation analysis was employed to assess the linear relationship between two variables. Survival time differences were assessed using the log-rank test. Significance levels are denoted by asterisks: ns for nonsignificant, * for p < 0.05, ** for p < 0.01, *** for p < 0.001, and **** for p < 0.0001. A p-value of less than 0.05 was set as the threshold for statistical significance.

## 3 Results

### 3.1 The radiosensitivity of LUAD is closely associated with glutamine metabolism

**Figure 1:**
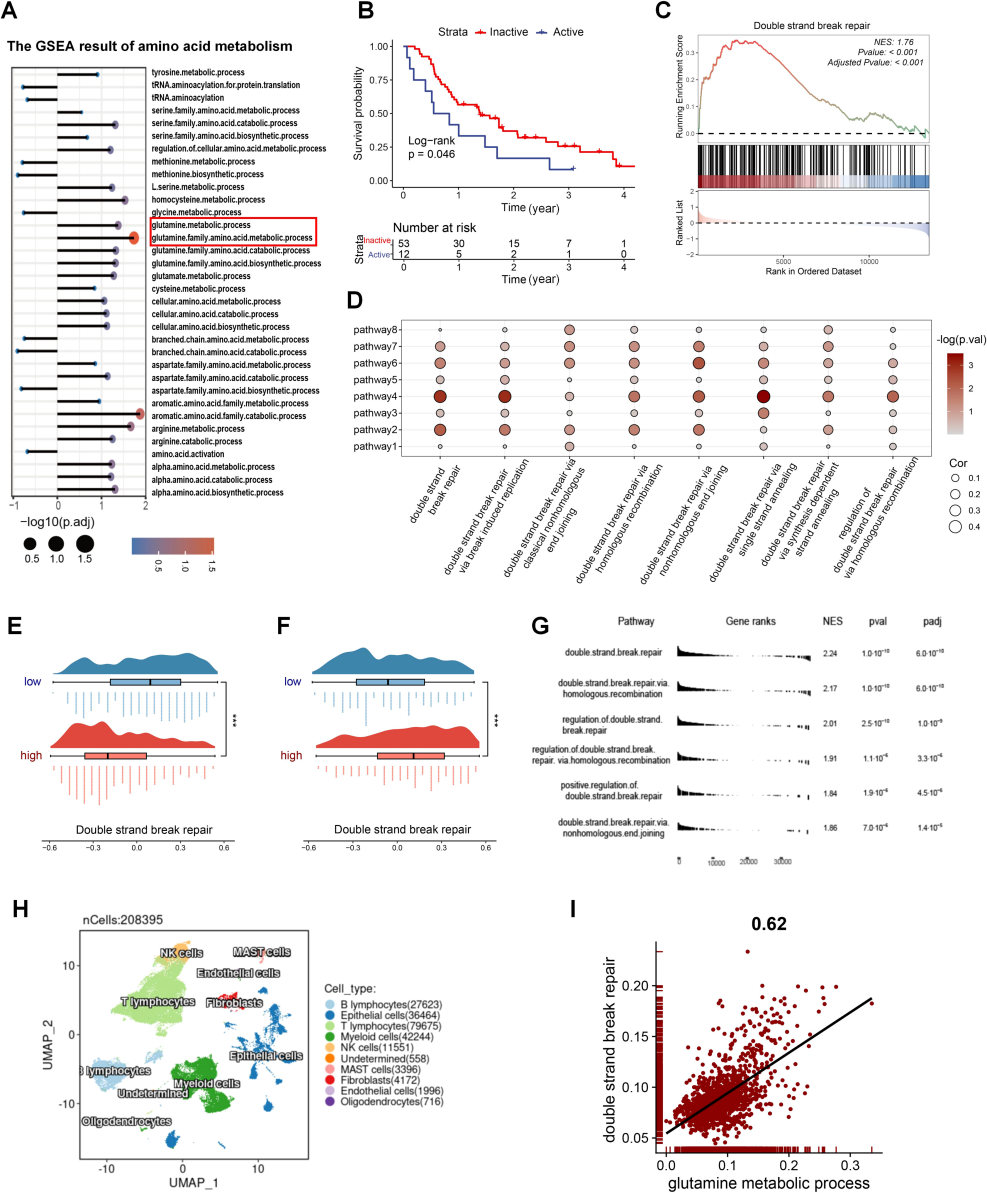
The radiosensitivity of LUAD is closely linked to glutamine metabolism. **(A)** GSEA results of amino acid metabolism pathways in radioresistant and radiosensitive patients. **(B)** The impact of glutamine metabolism on radiotherapy prognosis. **(C)** GSEA results of the DSB repair gene set in radioresistant and radiosensitive patients. **(D)** The correlation of glutamine-metabolism related pathways and DSB repair related pathways in patients with radiotherapy in GSE68456. Pathway1: glutamate metabolic process. Pathway2: glutamate biosynthetic process. Pathway3: glutamate catabolic process. Pathway4: glutamine metabolic process. Pathway5: glutamine family amino acid metabolic process. Pathway6: glutamine family amino acid catabolic process. Pathway 7: glutamine family amino acid biosynthetic process. Pathway8: regulation of glutamate metabolic process. **(E)** DSB repair activity between the two groups with different glutamine catabolic flux. A negative glutamine metabolic flux indicates active catabolism, with smaller values suggesting increased catabolic activity. **(F)** DSB repair activity between the two groups with different glutamate biosynthetic flux. A positive glutamate biosynthetic flux indicates active biosynthesis, with higher values suggesting increased biosynthetic activity. **(G)** GSEA results of the DSB repair related gene sets between varying glutamine catabolic flux in TCGA_LUAD. **(H)** Single-cell profile of GSE131907. **(I)** The correlation of glutamine metabolism and DSB repair in tumor cells of GSE131907. Wilcox rank-sum test was used for panel **(E, F)**. DSB, double strand break. Significance levels are denoted by asterisks: *** for p < 0.001.

Considering the impact of amino acid metabolism on radiosensitivity, we conducted GSEA on amino acid metabolic pathways in patients with varying radiosensitivity. The results showed significant enrichment of the glutamine metabolism pathway in the radioresistant group, suggesting that inhibiting glutamine metabolism may enhance radiosensitivity (**Figure 1A**; **Supplementary Figure S2A**). Subsequently, we assessed the glutamine metabolic flux in radiotherapy patients using Metaflux and performed Kaplan-Meier survival analysis, which demonstrated that lower glutamine catabolism was associated with improved survival (**Figure 1B**). We further analyzed the DSB repair pathway in the radiosensitive and radioresistant groups using GSEA, revealing significant enrichment of the DSB repair pathway in the radioresistant population (**Figure 1C**). To investigate the relationship between glutamine metabolism and DSB repair in radiotherapy populations, we performed GSVA on relevant pathways and conducted correlation analysis. The results revealed a strong association between them (**Figure 1D**), observed not only in the radiotherapy cohort (**Supplementary Figure S2B**). We also examined the relationship between glutamine, glutamate metabolic flux and DSB repair pathways. As shown in **Figures 1E, F**, patients with more active glutamine catabolism had higher DSB repair pathways scores, while those with more active glutamate biosynthesis exhibited lower DDR scores. In the radiotherapy population, individuals with active glutamine metabolism are significantly enriched in the DSB repair pathway (**Supplementary Figure S2C**). To additionally substantiate these findings, GSEA based differences in glutamine and glutamate metabolic flux revealed DSB repair pathways were significantly enriched in patients with more active glutamine catabolism and glutamate biosynthesis (**Figure 1G**; **Supplementary Figure S2D**). We analyzed a single-cell dataset to further investigate the relationship between glutamine metabolism and DSB repair in LUAD (**Figure 1H**) and extracted samples derived from lung tissue (**Supplementary Figure S3A**). After scoring the tumor cells for glutamine metabolic pathways and DSB repair, the analysis of correlation indicated a notably positive correlation between the two (**Figure 1I**). These findings demonstrate that inhibition of glutamine catabolism represents a potential therapeutic strategy to improve radiosensitivity.

**Figure 2:**
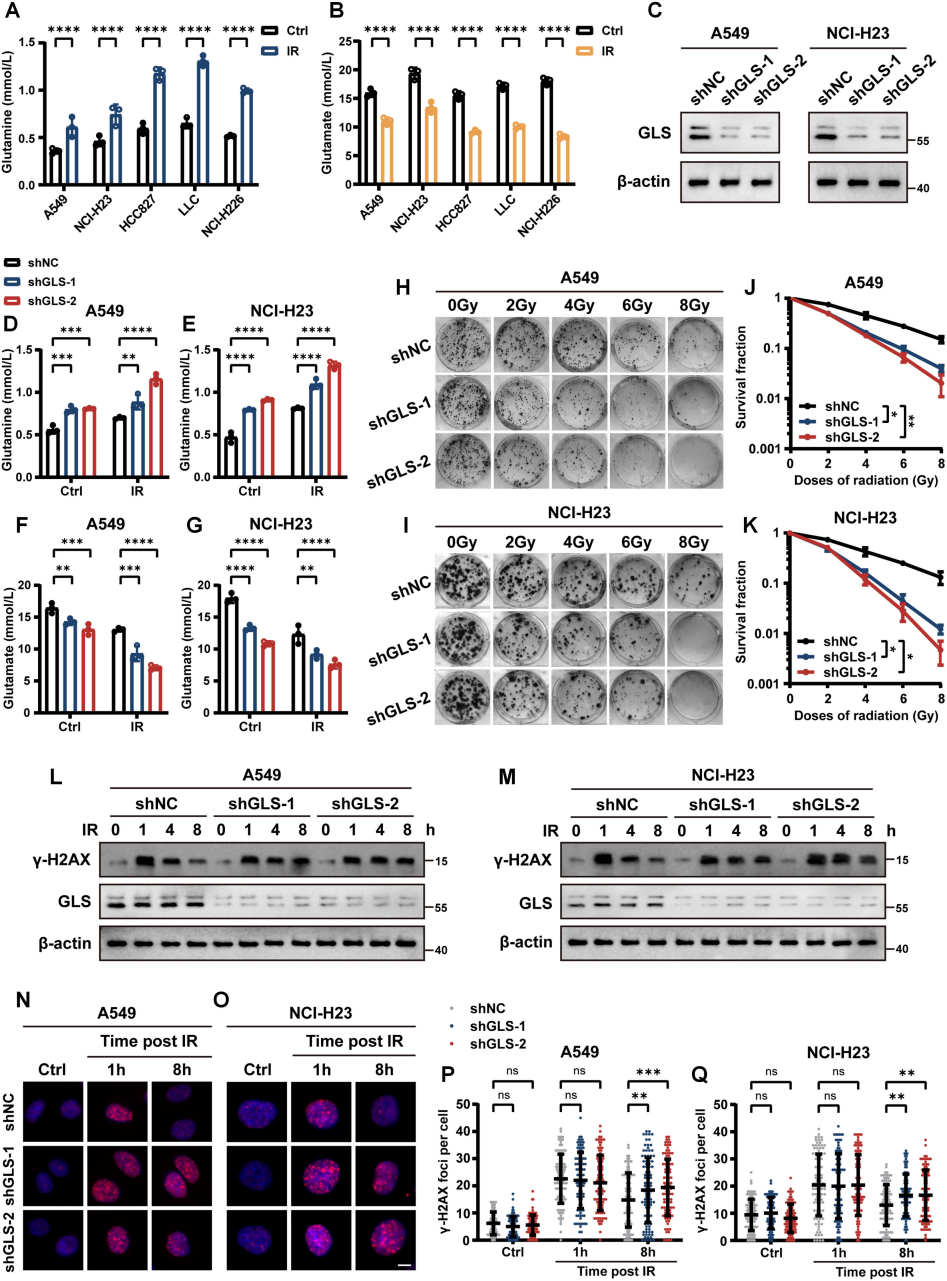
Knockdown of glutaminase enhanced radiosensitivity in LUAD cells *in vitro*. **(A, B)** Quantitative analysis of intracellular glutamine **(A)** and glutamate **(B)** levels in A549, NCI-H23, HCC827, LLC and NCI-H226 cells before and after irradiation. **(C)** Western blot confirming GLS knockdown efficiency in stably transfected A549 and NCI-H23 cells. **(D, E)** Intracellular glutamine levels were measured in control and GLS-knockdown A549 and NCI-H23 cells with or without exposure to ionizing radiation (10 Gy). **(F, G)** Intracellular glutamate levels were measured in control and GLS-knockdown A549 and NCI-H23 cells with or without exposure to ionizing radiation (10 Gy). **(H-K)** Representative images of colony formation assays in control and GLS-knockdown A549 and NCI-H23 cells exposed to various doses of ionizing radiation **(H, I)**. Survival curves derived from colony formation are presented in panel **(J, K)**. Data are presented as mean ± SEM (n = 3). **(L, M)** Western blot analysis of γ-H2AX protein levels in control and GLS-knockdown A549 and NCI-H23 cells following ionizing radiation (10 Gy) at 1, 4, and 8 hours, compared to untreated (0 h) conditions. **(N-Q)** Control and GLS-knockdown A549 and NCI-H23 cells were exposed to ionizing radiation (1 Gy), and γ-H2AX foci formation was assessed at specified time points. Representative images **(N, O)** and quantification of foci per nucleus **(P, Q)** are shown. Each group included counting over 100 cells. Scale bar, 10 μm. Data are presented as mean ± S.D. Data in panel **(A, B, D-G)** are presented as mean ± S.D (n = 3). Two-way ANOVA test was used for **(A, B, D-G, J, K, P, Q)**. IR, ionizing radiation; NC, negative control. Significance levels are denoted by asterisks: ns for nonsignificant, * for p < 0.05, ** for p < 0.01, *** for p < 0.001, and **** for p < 0.0001.

Then, we investigated the changes in intracellular glutamine and glutamate levels in five lung cancer cell lines before and after exposure to IR. The results indicated that IR led to an increase in intracellular glutamine levels, accompanied by a decrease in glutamate levels (**Figures 2A, B**). Since GLS catalyzes the conversion of glutamine to glutamate, it serves as a critical “node” regulating both metabolites in opposing directions. In order to further explore the influence of glutamine metabolism on radiotherapy efficacy, we performed GLS knockdown in A549 and NCI-H23 cells (**Figure 2C**). As expected, GLS silencing potentiated the IR-induced glutamine accumulation and concurrent glutamate reduction in LUAD cells (**Figures 2D–G**). To evaluate the effect of GLS on radiosensitivity in LUAD cells, we assessed the impact of GLS knockdown on colony formation and alteration of DNA damage marker γ-H2AX after IR. The colony formation assays demonstrated that GLS knockdown significantly decreased the surviving fraction of both A549 and NCI-H23 cells exposed to IR (**Figures 2H–K**). Western blot of γ-H2AX clearance following IR in A549 and NCI-H23 cells revealed a significantly decelerated decline in γ-H2AX levels in GLS knockdown cells compared to control cells over an 8-hour period (**Figures 2L, M**). Furthermore, Immunofluorescence (IF) staining confirmed that γ-H2AX foci in control cells nearly disappeared by 8 hours post-IR, but the foci remained detectable in GLS-knockdown cells at the same time point (**Figures 2N–Q**). Collectively, these results suggested that GLS knockdown effectively enhanced radiosensitivity of LUAD cells. Targeting glutamine metabolism, particularly through GLS inhibition, represents a potential strategy to enhance radiosensitivity in lung cancer therapy.

### 3.2 The role of GLS in reshaping the TME in LUAD

**Figure 3:**
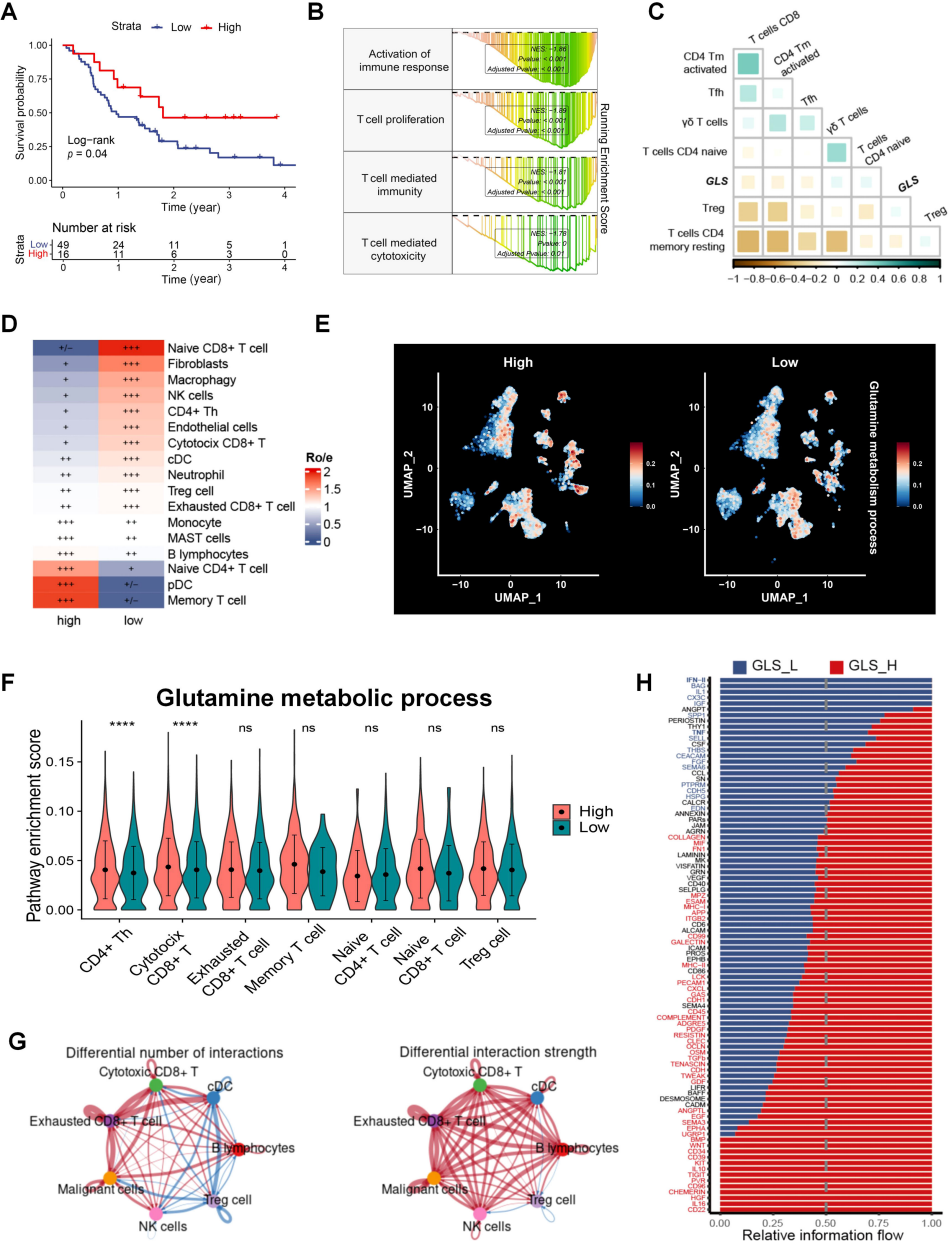
GLS is associated with the TME in LUAD. **(A)** The impact of CD8^+^ T cells on survival in the radiotherapy population in patients with radiotherapy in GSE68456. **(B)** GSEA results of the immune-related gene sets in GLS-low and GLS-high patients in GSE68456. **(C)** The correlation between the expression of GLS and T cell subpopulations. **(D)** Differences in immune cell distribution between GLS-high and GLS-low patients at the single-cell level. **(E)** Distribution of glutamine metabolic activity across different cell subpopulations between GLS-high and GLS-low groups. **(F)** Differences in glutamine metabolic activity across different T cell subpopulations between GLS-high and GLS-low groups. **(G)** Differences in the number and strength of interactions between immune cells and malignant cells between GLS-high and GLS-low groups. **(H)** Differences in the information flow of cellular interactions between the GLS-high and GLS-low groups. Wilcox rank-sum test was used for panel **(D)**. GLS_L, GLS-low; GLS_H, GLS-high. Significance levels are denoted by asterisks: ns for nonsignificant and **** for p < 0.0001.

Since the pivotal role of TME in the response of therapies in tumor, we evaluated immune cell infiltration in radiotherapy patients and found that CD8^+^ T cells significantly impact survival in patients (**Figure 3A**). Patients were classified into two groups depending on GLS expression, and GSEA results revealed that significant enrichment of the activation of immune responses in the GLS-low groups, including T cell proliferation and cytotoxicity (**Figure 3B**; **Supplementary Figure S3B**). The GLS expression and glutamine metabolism in radiotherapy patients were closely associated with immune cell infiltration (**Supplementary Figures S3C, D**), particularly with various T cell subpopulations, both of which exhibited an inverse correlation with CD8^+^ T cells (**Figure 3C**; **Supplementary Figures S3E–G**). In single-cell dataset, patients were categorized into two groups according to GLS expression in tumor cells, and the Ro/e analysis revealed significant differences in tumor cell infiltration between the groups (**Figure 3D**). Consistent with bulk RNAseq data, patients with lower GLS expression exhibited a significantly elevated infiltration of CD8^+^ T cells (**Figure 3D**). Further analysis of glutamine metabolism in these two groups showed that patients with higher GLS expression in tumor cells exhibited significant differences in glutamine metabolic activity in various immune cell types (**Figure 3E**; **Supplementary Figure S4A**). Additionally, analysis of T cell subpopulations indicated that in the high GLS group, the glutamine metabolism of cytotoxic T cells was notably more active (**Figure 3F**). Malignant cells in the high-GLS group also showed much greater glutamine metabolism than those in the GLS-low group (**Supplementary Figure S4B**). CellChat analysis further revealed significant differences in the quantity and strength of cell-cell communication between the high- and low- GLS groups (**Figure 3G**; **Supplementary Figure S4C**). In the GLS-low group, the interplay between cytotoxic T cells and malignant cells was stronger compared to the high GLS group. Further analysis of intercellular signaling pathways in the two patient groups revealed significant differences, with pathways such as the type II interferon (IFN) pathway and the tumor necrosis factor (TNF) pathway exhibiting notably higher activation in the low GLS group than in the high GLS group (**Figure 3H**). These findings suggested that the GLS-low group exhibited a more potent cytotoxic effect of T cells on tumor cells. To further investigate the interaction between malignant cells with different GLS levels and effector T cells, we analyzed two spatial transcriptomic samples from LUAD at the same stage (**Supplementary Figures S5A, B**). The results indicated that in samples with lower GLS expression in malignant cells, the exclusion coefficient of co-localization between malignant cells and effector T cells was lower, suggesting a greater likelihood of effector T cell infiltration into the tumor (**Supplementary Figures S5C, D**). In contrast, the exclusion coefficient with Tregs was higher, indicating a reduced likelihood of Tregs infiltration, which in turn was more favorable for effector T cells to exert their tumor-killing functions (**Supplementary Figure S5E**). The coefficient for co-localization between effector T cells and Tregs was negative in GLS-low samples and positive in GLS-high samples, indicating an inhibitory effect on effector T cells in GLS-high samples (**Supplementary Figures S5F–H**).

**Figure 4:**
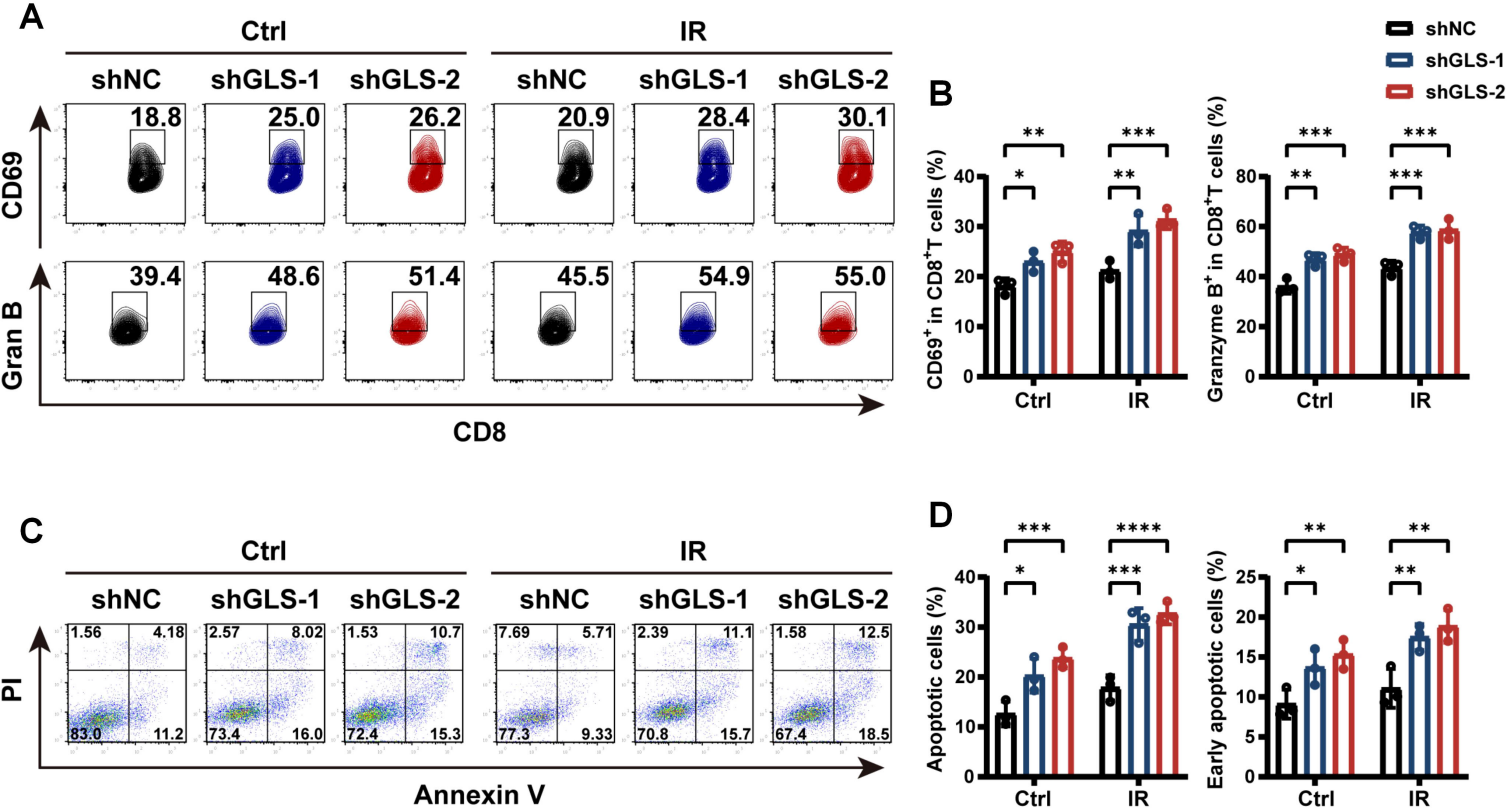
GLS knockdown promotes CD8^+^ T cell-mediated tumor-killing. **(A, B)** Control and GLS-knockdown A549 cells, with or without exposure to 10 Gy ionizing radiation, were co-cultured with human PBMCs. After 1 and 3 days respectively, the proportions of CD69^+^ and Granzyme B^+^ cells among CD8^+^ T cells were assessed by flow cytometry. Representative flow cytometry plots **(A)** and quantification of positive cell rates **(B)** are shown. **(C, D)** Control and GLS-knockdown A549 cells, with or without exposure to 10 Gy ionizing radiation, were co-cultured with human PBMCs. After 12 h, apoptosis assay with Annexin V-APC/PI staining of tumor cells was performed using flow cytometry. Representative images were shown in panel **(C)**. Percent apoptotic cells (Annexin V^+^) and early apoptotic cells (Annexin V^+^ PI^-^) were quantified in panel **(D)**. All data are presented as mean ± S.D (n = 3). Two-way ANOVA test was used for panel **(B, D)**. IR, ionizing radiation; NC, negative control; Gran B, Granzyme B, PBMCs, peripheral blood mononuclear cells; PI, Propidium Iodide. Significance levels are denoted by asterisks: * for p < 0.05, ** for p < 0.01, *** for p < 0.001, and **** for p < 0.0001.

Considering the critical role of CD8^+^ T cells in antitumor immune responses following radiotherapy, we conducted a co-culture assay using PBMCs to validate the impact of GLS on CD8^+^ T cell activation and cytotoxicity. The GLS-knockdown A549 cells or control cells with or without IR were co-cultured with human PBMCs. The results demonstrated that GLS knockdown markedly raised the percentage of CD8^+^ T cells that express the activation marker CD69 after 24 hours of co-culture (**Figures 4A, B**). After 72 hours of stimulation, the percentage of CD8^+^ T cells with Granzyme B expression, a crucial marker of cytotoxic activity, was markedly higher in the GLS knockdown group (**Figures 4A, B**). In addition, within the co-culture system, A549-shGLS cells exhibited a significantly higher apoptosis rate compared to control cells, further demonstrating that GLS inhibition enhances the tumor-killing effect of immune cells (**Figures 4C, D**). These findings suggest that downregulation of GLS enhances the immune response against LUAD cells, primarily by promoting the activation and cytotoxic function of CD8^+^ T cells in the context of radiotherapy.

### 3.3 Knockdown of GLS promotes ferroptosis, thereby increasing cellular death following IR treatment

**Figure 5:**
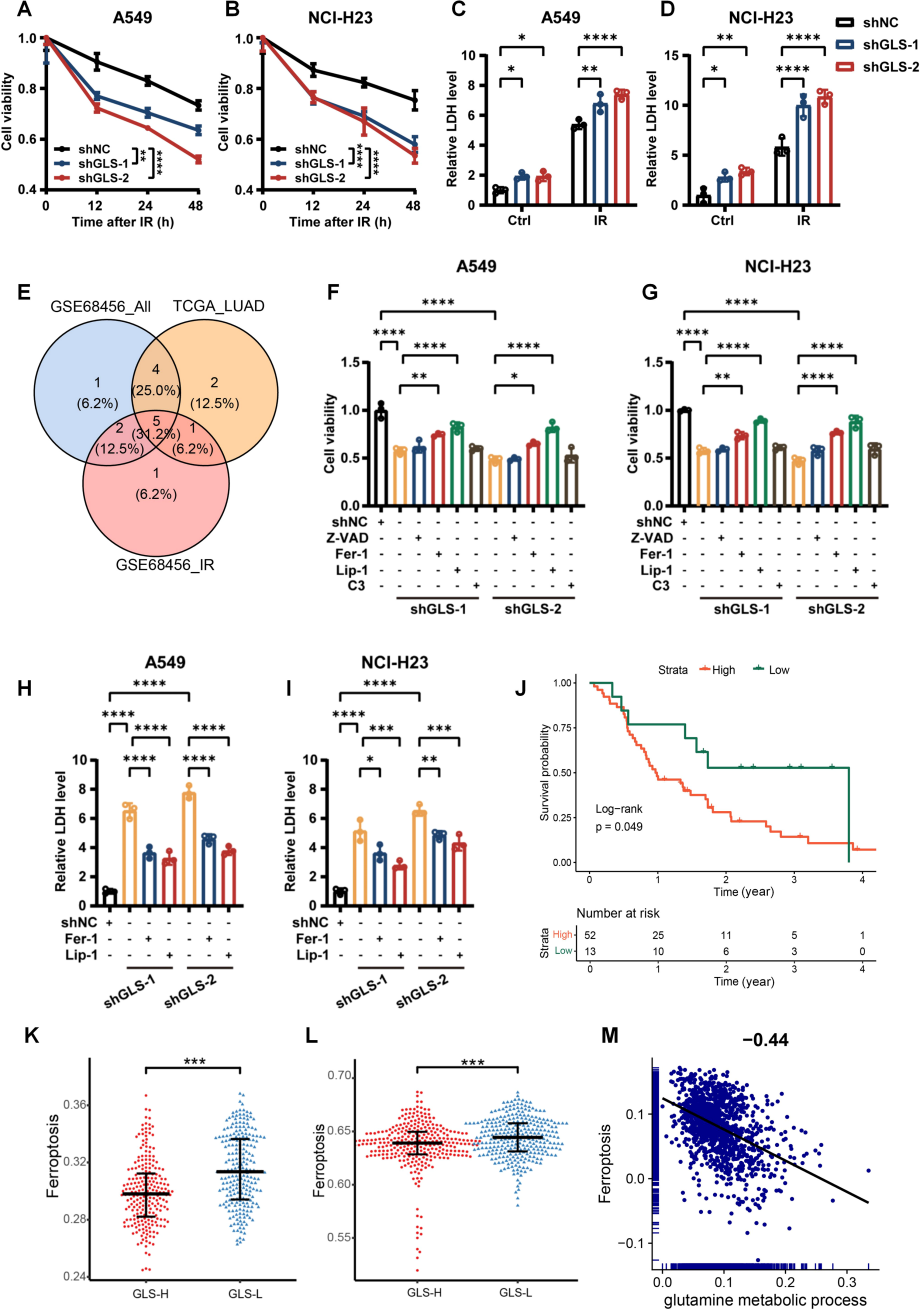
GLS depletion promotes irradiation-induced cell death via ferroptosis in LUAD cells. **(A, B)** The cell viability curves of control and GLS-knockdown A549 and NCI-H23 cells at 12 h, 24 h and 48 h after ionizing radiation (10 Gy). **(C, D)** The lactate dehydrogenase release levels from control and GLS-knockdown A549 and NCI-H23 cells with or without exposure to ionizing radiation (10 Gy). **(E)** The types of PCD associated with prognosis in radiotherapy patients and correlated with GLS in both GSE68456 and TCGA_LUAD. **(F, G)** GLS-knockdown A549 and NCI-H23 cells were pre-treated for 24 hours with inhibitors of ferroptosis (Fer-1, Lip-1), apoptosis (Z-VAD), or entosis (C3 exoenzyme) prior to exposure to IR (10 Gy). Cell viability was subsequently assessed using the CCK-8 assay. **(H, I)** GLS-knockdown A549 and NCI-H23 cells were pre-treated for 24 hours with inhibitors of ferroptosis (Fer-1, Lip-1) prior to exposure to IR (10 Gy). Lactate dehydrogenase release from cells was evaluated. **(J)** The impact of ferroptosis on the outcomes of patients receiving radiotherapy. **(K, L)** Differences in ferroptosis between GLS-high and GLS-low groups in GSE68456 **(K)** and TCGA_LUAD **(L)**. **(M)** The correlation of glutamine metabolism and ferroptosis in tumor cells of LUAD patients. All data are presented as mean ± S.D (n = 3). Two-way ANOVA test was used for **(A-D)**; One-way ANOVA test was used for **(F-I)**. Wilcox rank-sum test was used for **(K, L)**. IR, ionizing radiation; NC, negative control; Z-VAD, Benzyloxycarbonyl-Val-Ala-Asp fluoromethyl ketone; Fer-1, Ferrostatin-1; Lip-1, Liproxstatin-1; C3, C3 Exoenzyme; LDH, Lactate Dehydrogenase; PCD, programmed cell death. Significance levels are denoted by asterisks: * for p < 0.05, ** for p < 0.01, *** for p < 0.001, and **** for p < 0.0001.

Radiotherapy induces tumor cell death through various mechanisms. To investigate the impact of GLS knockdown on the mode of death in irradiated cells, we first assessed cell viability and their LDH release. As illustrated in **Figures 5A–D**, silencing GLS in A549 and NCI-H23 cells following IR resulted in a reduction in cell viability and a rise in LDH release. To explore the specific type of cell death induced by GLS knockdown that sensitize tumors to radiotherapy, we analyzed 18 types of PCD in relation to radiotherapy outcomes and GLS expression. We intersected the PCDs associated with radiotherapy response with those found to be correlated with GLS expression in both the TCGA_LUAD and GSE68456 datasets, identifying five types of PCD, including apoptosis, ferroptosis, unprogrammed necrosis, and entosis (**Figure 5E**). Next, we pretreated the cells with pharmacological inhibitors targeting various cell death pathways. The results showed that two distinct ferroptosis inhibitors, Fer-1 and Lip-1, effectively rescued GLS-depleted A549 and NCI-H23 cells from IR-induced cell death. In contrast, inhibitors of apoptosis (Z-VAD) and entosis (C3 exoenzyme) had only a slight effect (**Figures 5F, G**). Consistently, treatment with Fer-1 and Lip-1 significantly alleviated LDH release from GLS-knockdown A549 and NCI-H23 cells post-irradiation (**Figures 5H, I**). Survival analysis suggests that ferroptosis has a significant impact on the survival of patients undergoing radiotherapy (**Figure 5J**). Ferroptosis varies greatly between groups with high and low GLS expression (**Figures 5K, L**). Single-cell data further reveal a significant negative correlation between glutamine metabolism and ferroptosis in tumor cells (**Figure 5M**). These results suggested that the reduced viability observed in GLS-silenced LUAD cells following irradiation was primarily attributable to ferroptosis.

**Figure 6:**
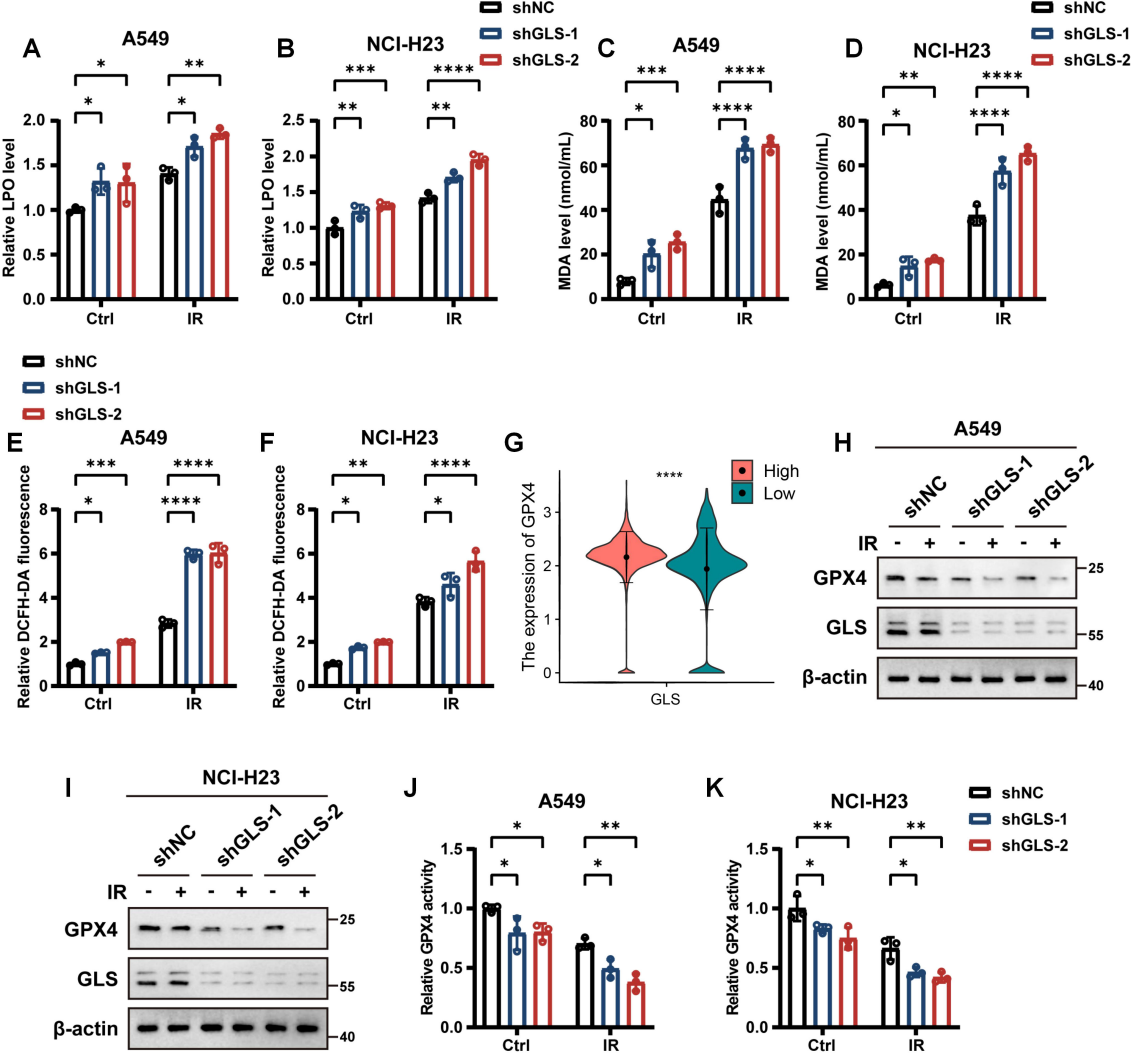
Inhibition of GLS promotes ferroptosis in LUAD cells. **(A-D)** Relative lipid peroxidation levels **(A, B)** and quantification of malondialdehyde levels **(C, D)** in control and GLS-knockdown A549 and NCI-H23 cells with or without exposure to ionizing radiation (10 Gy). **(E, F)** Intracellular ROS levels detected by DCFH-DA fluorescent probe in control and GLS-knockdown A549 and NCI-H23 cells with or without exposure to ionizing radiation (10 Gy). **(G)** The expression of GPX4 in malignant cells of GLS-high and GLS-low patients at the single-cell level. **(H, I)** Western blot analysis of GPX4 protein levels in control and GLS-knockdown A549 and NCI-H23 cells with or without exposure to ionizing radiation (10 Gy). **(J, K)** Relative GPX4 activity in control and GLS-knockdown A549 and NCI-H23 cells with or without exposure to ionizing radiation (10 Gy). All data are presented as mean ± S.D (n = 3). Wilcox rank-sum test was used for panel **(G)**. Two-way ANOVA test was used for panel **(A-F)**, **(J, K)**. IR, ionizing radiation; NC, negative control; LPO, lipid peroxidation; MDA, malondialdehyde; DHE, dihydroethidium; DCFH-DA, 2’,7’-Dichlorodihydrofluorescein Diacetate; GPX4, glutathione peroxidase 4; ROS, reactive oxygen species. Significance levels are denoted by asterisks: * for p < 0.05, ** for p < 0.01, *** for p < 0.001, and **** for p < 0.0001.

To further investigate the contribution of ferroptosis in the increased radiosensitivity of GLS-depleted LUAD cells, we assessed several ferroptosis-related indicators. LPO, a hallmark of ferroptosis, is primarily indicated by the accumulation of its final product, MDA. As shown in **Figures 6A–D**, the levels of both LPO and MDA were significantly elevated in GLS-depleted LUAD cells. Intracellular ROS, a major inducer of lipid peroxidation, were quantified using the cytosolic ROS sensors DCFH-DA and DHE. As expected, GLS silencing notably increased intracellular ROS levels in A549 and NCI-H23 cells following irradiation (**Figures 6E, F**; **Supplementary Figure S6**). Moreover, comparison between GLS-high and GLS-low patients revealed significantly elevated GPX4 expression in malignant cells (**Figure 6G**). GPX4, a critical enzyme that mitigates lipid peroxidation, is essential for regulating ferroptosis (46, 47). Western blot and GPX4 activity assays confirmed that GLS knockdown reduced both the expression and activity of GPX4 in NSCLC cells (**Figures 6H–K**). In conclusion, GLS knockdown enhances ferroptosis via downregulating GPX4, thereby enhancing LUAD cell death upon IR treatment.

### 3.4 GLS-knockdown potentiates the radiosensitivity of LUAD cells *in vivo*

**Figure 7:**
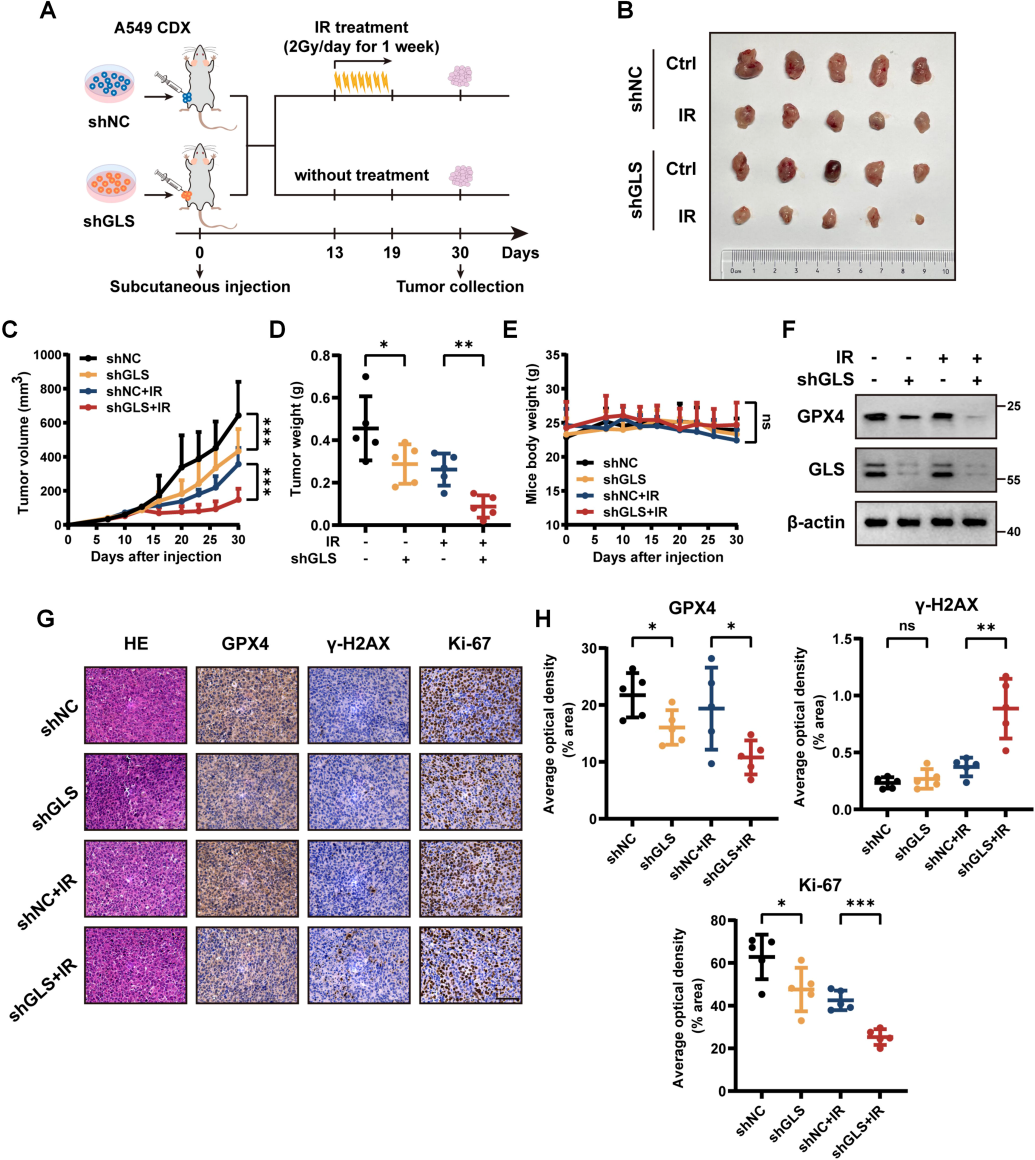
Inhibition of GLS sensitizes LUAD to radiotherapy *in vivo*. **(A)** Schematic diagram of the radiotherapy xenograft model. A549 cells stably expressing shNC or shGLS were subcutaneously injected into nude mice. Once tumor volumes reached 60-120 mm³, mice bearing control or GLS-knockdown tumors were randomized into irradiated and non-irradiated groups (n = 5). Mice in the irradiated group received 14 Gy radiation over one week (2 Gy/day). **(B)** Photographs of tumors in each group were taken on day 30. **(C-E)** Tumor growth curves **(C)**, tumor weights **(D)**, and body weight changes of mice **(E)** in each group (n = 5). **(F)** Western blot analysis of GPX4 in resected tumor samples from each group. **(G, H)** Representative images **(G)** and quantitative data **(H)** of IHC for GPX4, γ-H2AX and Ki-67 in tumor samples from control and GLS-knockdown group with or without radiotherapy (n = 5). Representative images of HE staining for tumors in each group are also shown in panel **(G)**. Scale bar, 50 μm. All data are presented as mean ± S.D. Two-way ANOVA test was used for panel **(C-E)**; Two-tailed unpaired t test was used for panel **(H)**. IR, ionizing radiation; NC, negative control; CDX, cell-derived xenograft; GPX4, glutathione peroxidase 4; HE, hematoxylin and eosin; IHC, immunohistochemistry. Significance levels are denoted by asterisks: ns for nonsignificant, * for p < 0.05, ** for p < 0.01 and *** for p < 0.001.

To further confirm these findings *in vivo*, we subcutaneously implanted control and GLS-knockdown A549 cells into BALB/c nude mice to create xenograft tumors (**Figure 7A**). Consistent with *in vitro* findings, GLS silencing significantly enhanced radiosensitivity in LUAD subcutaneous xenograft models, as evidenced by reductions in tumor volume and weight following radiotherapy (**Figures 7B–D**). No notable variations in body weight were recorded among the experimental groups (**Figure 7E**). Additionally, Western blot and IHC analysis of tumor tissues revealed a marked reduction in GPX4 expression in the GLS-knockdown group, suggesting an enhanced level of ferroptosis (**Figures 7F–H**). IHC analysis demonstrated markedly reduced Ki-67 expression and elevated γ-H2AX levels in the GLS-knockdown group following IR treatment, indicating an augmented anti-tumor efficacy (**Figures 7G, H**). These results collectively demonstrate that GLS depletion sensitizes LUAD cells to radiotherapy *in vivo*.

### 3.5 Establishment of the GLS-DSBr prognostic model

**Figure 8:**
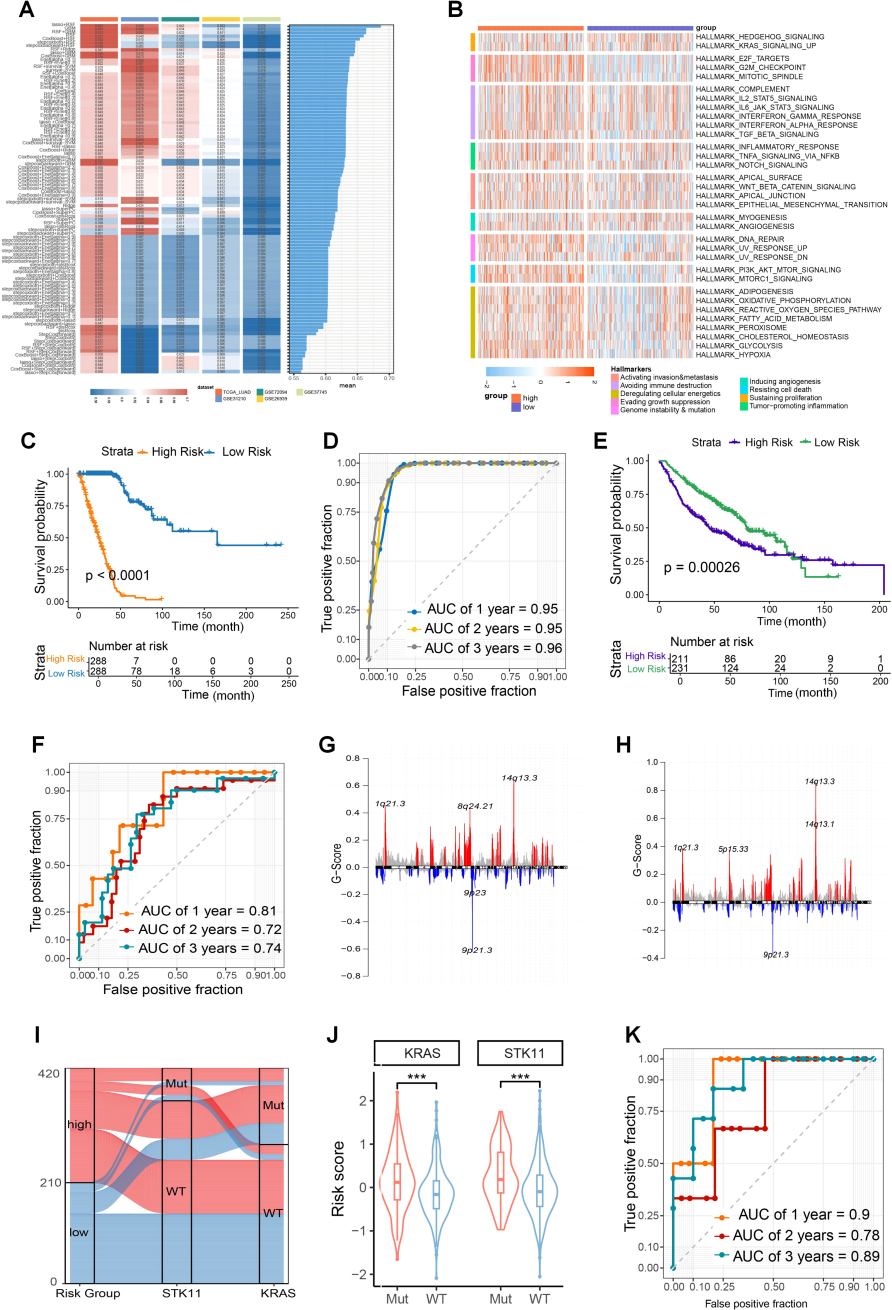
Construction and functional exploration of the GLS-DSBr Model. **(A)** C-index of 101 machine learning algorithm models in LUAD datasets. **(B)** The result of Hallmark analysis in high- and low-risk groups. **(C)** Kaplan-Meier survival analysis based on GLS-DSBr score in TCGA_LUAD. **(D)** The ROC curve of GLS-DSBr model in TCGA_LUAD. **(E)** Kaplan-Meier survival analysis based on GLS-DSBr score in GSE68456. **(F)** The ROC curve of GLS-DSBr model in population with radiotherapy. **(G, H)** CNV characteristics in high- and low-risk groups. **(I)** Sankey diagram of STK11 and KRAS mutations in high- and low-risk groups. **(J)** Risk scores between STK11 and KRAS wild-type and mutant populations. **(K)** The ROC curve of GLS-DSBr model in patients with immunotherapy of GSE13522. Wilcox rank-sum test was used for panel **(J)**. Significance levels are denoted by asterisks: *** for p < 0.001.

Given the association between glutamine metabolism and DSB repair with tumor malignancy, we explored their combined value in predicting tumor prognosis. Using the TCGA_LUAD dataset as the training cohort, along with four independent validation cohorts (GSE31210, GSE72094, GSE50081, and GSE37745), we constructed a prognostic model based on the intersection of differentially expressed genes stratified by GLS expression and the DSB repair gene set. First, univariate Cox regression was performed (**Supplementary Table S1**), followed by machine learning methods. Selection of the optimal model was guided by the average C-index (**Figure 8A**; **Supplementary Figures S7A, B**). GSVA analysis of Hallmark gene sets revealed that the low-risk group exhibited significantly lower activity in key pathways, including DNA repair and energy metabolism, compared to the high-risk group (**Figure 8B**). According to the risk scores obtained from the GLS-DSBr model, we plotted Kaplan-Meier curves. The Log-rank test indicated statistically significant differences in survival between the two groups across all datasets (**Figure 8C**; **Supplementary Figures S7C–F**). In addition, the ROC curves revealed that the GLS-DSBr model offers superior prognostic predictive performance (**Figure 8D**; **Supplementary Figures S7G–J**). When applied to the GSE68456 dataset, the model effectively differentiated patients with distinct prognosis (**Figure 8E**) and presented robust predictive power for the radiotherapy population, with AUC values of approximately 0.8 at 1, 2, and 3 years (**Figure 8F**).

At the genomic level, there were differences in CNVs between the high- and low-risk groups. Specifically, the high-risk group exhibited a stronger gain at 1q21.3 and a more pronounced loss at 9p21.3 compared to the low-risk group (**Figures 8G, H**). The overall SNP mutation frequency was higher in the high-risk group, the top 10 genes with the highest incidence of SNPs, which showed obvious differences between the groups in the TCGA-LUAD cohort (**Supplementary Figure S8A, B**).

In the GSE72094 dataset, high-risk patients showed a higher proportion of KRAS and STK11 mutations (**Figures 8I, J**). Given the association of STK11 with immunotherapy response in LUAD patients, we performed risk assessment using the GSE135222 dataset. The results indicated that GLS-DSBr score was strongly correlated with survival in LUAD patients receiving anti-PD-1/PD-L1 therapy, with excellent prognostic prediction ability (1-, 2-, and 3-year AUC values of 0.9, 0.78, and 0.89, respectively) (**Figure 8K**; **Supplementary Figure S7K**).

**Figure 9:**
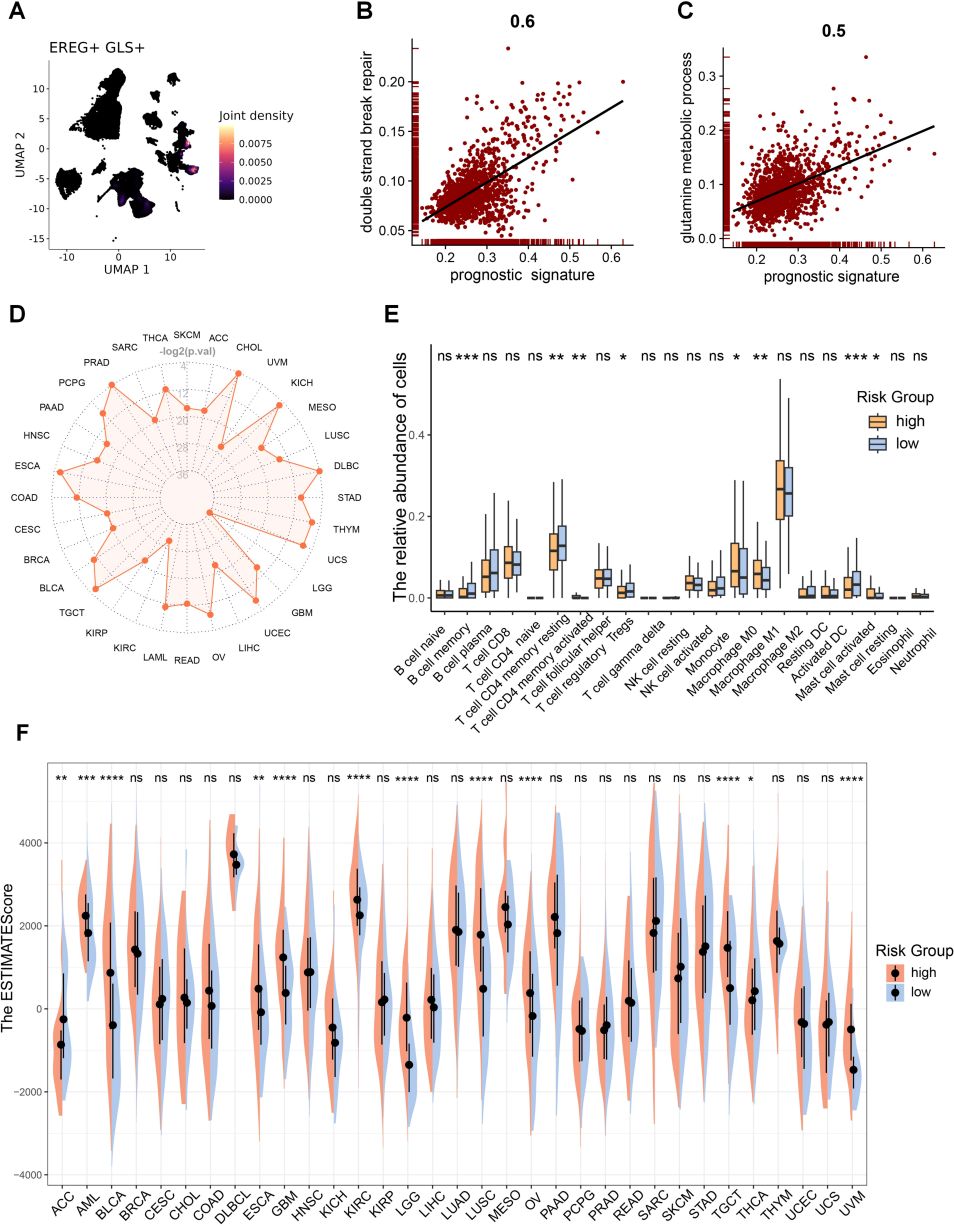
GLS-DSBr model is applicable across pan-cancer. **(A)** Co-expression of representative prognostic gene (EREG) with GLS. **(B)** Correlation between risk score and DSB repair in tumor cells. **(C)** Correlation between risk score and glutamine metabolism in tumor cells. **(D)** Radar plot of log-rank test p-values for the prognostic model across pan-cancer. -log2(0.05) = 4.32, -log2(0.01) = 6.64. **(E)** Differences in immune cell infiltration between RS-high and RS-low patients in LUAD. **(F)** Differences in TME between high-risk and low-risk patients in pan-cancer. Wilcox rank-sum test was used for panel **(E, F)**. RS, risk score. Significance levels are denoted by asterisks: ns for nonsignificant, * for p < 0.05, ** for p < 0.01, *** for p < 0.001, and **** for p < 0.0001.

The correlation between model genes and GLS was shown in **Supplementary Figure S8C**. EREG, exhibiting the strongest positive correlation with GLS, is predominantly co-expressed with GLS in epithelial cells (**Figure 9A**), while INSL4, showing the strongest negative correlation, is mainly co-expressed with GLS in myeloid cells (**Supplementary Figure S8D**). The GLS-DSBr score is positively correlated with DSB repair in tumor cells (**Figure 9B**), consistent with reports linking stronger DSB repair activity to higher tumor malignancy. Additionally, the GLS-DSBr score correlates positively with glutamine metabolism (**Figure 9C**), supporting the observation that reduced glutamine metabolism in tumor cells increases their susceptibility to cell death. Finally, a significant negative correlation was observed between the GLS-DSBr score and ferroptosis (**Supplementary Figure S8E**) in tumor cells. Collectively, the GLS-DSBr model establishes a clinically actionable framework that demonstrates robust prognostic accuracy and offers potential to guide personalized therapeutic strategies in LUAD, particularly for optimizing radiotherapy and immune checkpoint blockade efficacy.

### 3.6 The GLS-DSBr model is applicable across pan-cancer and correlates with the TME

Given that DSB repair and metabolic reprogramming are essential characteristics of cancer, A correlation analysis was carried out to examine the relationship between GLS expression and immune cell infiltration across pan-cancer. The analysis showed a notable association between GLS and the TME in the majority of cancers (**Supplementary Figure S9A**). Therefore, we examined the applicability of the GLS-DSBr model across pan-cancer. The results demonstrated that the GLS-DSBr model is applicable to nearly all cancer types (**Figure 9D**; **Supplementary Figure S9B**).

Further TME analysis between the two groups exhibited considerable disparities in the infiltration of immune cells (**Figure 9E**; **Supplementary Figure S10A**). To evaluate the TME landscape, we performed pan-cancer ESTIMATE analysis, which revealed significant differences in immune infiltration and stromal components between the two groups across most cancer types (**Figure 9F**; **Supplementary Figures S10B, C**). These results collectively suggest that the GLS-DSBr model not only holds broad applicability across pan-cancer but also reflects distinct immune and stromal features within the TME.

## 4 Discussion

In this study, we found that glutamine metabolism is closely associated with DSB repair, and knocking down GLS increased tumor sensitivity to radiotherapy. Further analysis revealed that GLS knockdown not only promoted ferroptosis in tumor cells but also enhanced the cytotoxicity of CD8^+^ T cells in TME. Our findings underscored the pivotal role of GLS in shaping the metabolic and immune landscapes of LUAD. Additionally, based on GLS and DSB repair, we applied machine learning to build a robust model for the prognosis and the efficacy of radiotherapy and immunotherapy in LUAD, which was validated for its applicability across pan-cancer.

Metabolic reprogramming is a key feature of tumor, significantly contributing to tumor progression and resistance to therapy (48). Cancers mostly exhibit a strong affinity for glutamine, and the metabolic dependence of tumor cells on glutamine has now been recognized as a distinctive phenomenon referred to as “glutamine addiction” (49). Our analysis suggests that glutamine metabolic flux is a key determinant of radiotherapy outcomes in LUAD. Unrepaired DSB are among the most detrimental forms of cellular damage, often leading to cell death (50). Enhanced DSB repair capacity is a major contributor to radioresistance (51, 52), and our correlation analysis revealed a strong link between DSB repair and glutamine metabolism.

IR induces cell death through various forms of PCD by damaging cellular DNA. Different forms of PCD are closely associated with tumorigenesis and therapeutic response (53). Our analysis of 18 forms of PCD revealed five types, including ferroptosis, which are significantly correlated with the radiotherapy sensitivity of LUAD and GLS. Glutathione (GSH), a major downstream product of glutamine metabolism, plays a critical role in radiation-induced ferroptosis (54, 55). Given the role of GSH in ferroptosis, we pre-treated shGLS cells with ferroptosis inhibitors before radiotherapy and found that the inhibitors largely prevented the increased radiotherapy sensitivity caused by GLS knockdown. This suggested that the enhanced cell death induced by GLS knockdown in radiotherapy is primarily ferroptosis. Single-cell analysis further indicated that glutamine metabolism in malignant cells is negatively correlated with ferroptosis, and lower GLS in malignant cells is accompanied by decreased GPX4. Experiments confirmed that shGLS cells showed significant reduction in GPX4 expression after radiotherapy. Previous studies have reported that tumor cells can develop adaptive defenses against ferroptosis induced by radiation, such as upregulating key inhibitory molecule like GPX4 (26). Our results suggested that GLS knockdown reverses the defense by downregulating GPX4, thereby enhancing radiosensitivity and overcoming radioresistance.

Other than malignant cells, immune cells also depend on energy to mount effective immune responses (56). However, nutrient availability, including glutamine, is restricted due to competition between various cell populations in TME (57). The activation of memory CD8^+^ T cells to boost cytotoxicity and cytokine production depends on glutamine (58). A deficiency in glutamine not only impairs the activation of effector T cells but also increases the infiltration of immunosuppressive Treg cells (59). GSEA analysis suggests that low GLS is associated with enhanced immune responses, particularly the cytotoxicity of T cells. The co-culture assay with PBMC validated that GLS knockdown significantly increased CD8^+^ T cell activation and cytotoxicity after IR. Therefore, we hypothesize that tumor cells with low GLS expression, due to reduced glutaminase activity, increase the glutamine in the TME, thereby alleviating glutamine deficiency in immune cells. Single-cell RNA sequencing revealed that low GLS expression increased the cytotoxicity of CD8^+^ T cells against malignant cells and decreased interaction of cytotoxic CD8^+^ T cells and Treg. Spatial transcriptomics analysis indicated that in patients with low GLS expression, the TME exhibits a higher abundance of effector T cell infiltration, while the infiltration of Treg cells is less. Depriving glutamine hinders secretion of IFN-γ by activated T cells (22). Moreover, ferroptosis can act as an immunogenic cell death pathway, releasing damage-associated molecular patterns (DAMPs) and lipid peroxidation products that further activate CD8^+^ T cells and enhance anti-tumor immunity (60, 61). This dual effect of GLS knockdown—directly inducing ferroptosis and indirectly modulating T cell activity—creates a synergistic effect that enhances the overall anti-tumor immune response. Supporting this hypothesis, we observed increased activity of the IFN-γ and TNF signaling pathways in samples with low GLS expression in malignant cells. Therefore, we propose that low GLS expression in malignant cells boosts effector T cell activation via IFN-γ and TNF signaling pathways, while reducing the inhibitory signals typically mediated by Treg cells. This shift in the TME enhances antitumor immunity by promoting T-cell activity and weakening Treg suppression, improving the overall immune response against the tumor.

Glutamine metabolism is closely associated with tumorigenesis, treatment response, and prognosis (13). DSB repair capacity is a classic hallmark of cancer and also closely linked to tumor progression (62). Considering the link among GLS, radiotherapy sensitivity, and DSB repair, we constructed a GLS_DSBr prognostic model developed through machine learning, trained and validated using data from 2066 patients. This model accurately predicted the prognosis of LUAD patient, as well as their response to radiotherapy and immunotherapy. In addition, we validated this model’s applicability across multiple cancer types. It not only showed excellent prognostic prediction performance in most cancer types but also revealed notable variations in the TME exist between low- and high-risk groups.

Although clinical trials of GLS inhibitors (e.g., NCT02861300, NCT03428217) have demonstrated safety, their efficacy remains heterogeneous and inconclusive. Further development of combinatorial therapeutic strategies and optimization of treatment regimens are imperative to realize clinical benefits in the future. Our findings address this challenge by identifying specific mechanisms (DSB repair suppression, ferroptosis induction, and TME modulation) that synergize GLS inhibition with radiotherapy and immunotherapy. Furthermore, the GLS-DSBr model provides a biomarker-driven framework to stratify patients likely to benefit from GLS-targeted therapies. Even so, several limitations should be acknowledged. First, while we demonstrate that GLS knockdown enhances radiosensitivity via promoting ferroptosis and improving immune activation in pre-clinical experiments, clinical validation of these findings in human patients is necessary. Although our machine learning model provides promising results, further validation in real-world cohorts is essential to fully confirm its clinical utility.

## 5 Conclusion

In conclusion, our study demonstrated that GLS inhibition suppresses glutamine catabolism, promoting ferroptosis in irradiated tumors, thus improving radiosensitivity. Moreover, the TME with low GLS expression amplifies the cytotoxicity of effector T cells against tumor cells, contributing to enhanced irradiation-induced immune response. Additionally, we developed a robust prognostic model, GLS-DSBr, capable of accurately predicting prognosis and the efficacy of both radiotherapy and immunotherapy in LUAD, with applicability across pan-cancer. These findings provide valuable insights into the metabolic regulation of cancer therapy, offering a promising strategy for improving patient outcomes in precision medicine.

## Data Availability

Publicly available datasets were analyzed in this study. This data can be found here: GSE31210, GSE72094, GSE50081, GSE37745, GSE68456, GSE131907, GSE135222, GSE189487.
