# Supplementary material for: The suppressive role of GLS in radiosensitivity and irradiation-induced immune response in LUAD: integrating bioinformatics and experimental insights
Source: Front Immunol. 2025 Apr 16;16:1582587. doi: 10.3389/fimmu.2025.1582587 (PMC12040943; doi:10.3389/fimmu.2025.1582587)
Supplement: Supplementary file 1 [file DataSheet1.pdf]

Figure S1. Graphical abstract

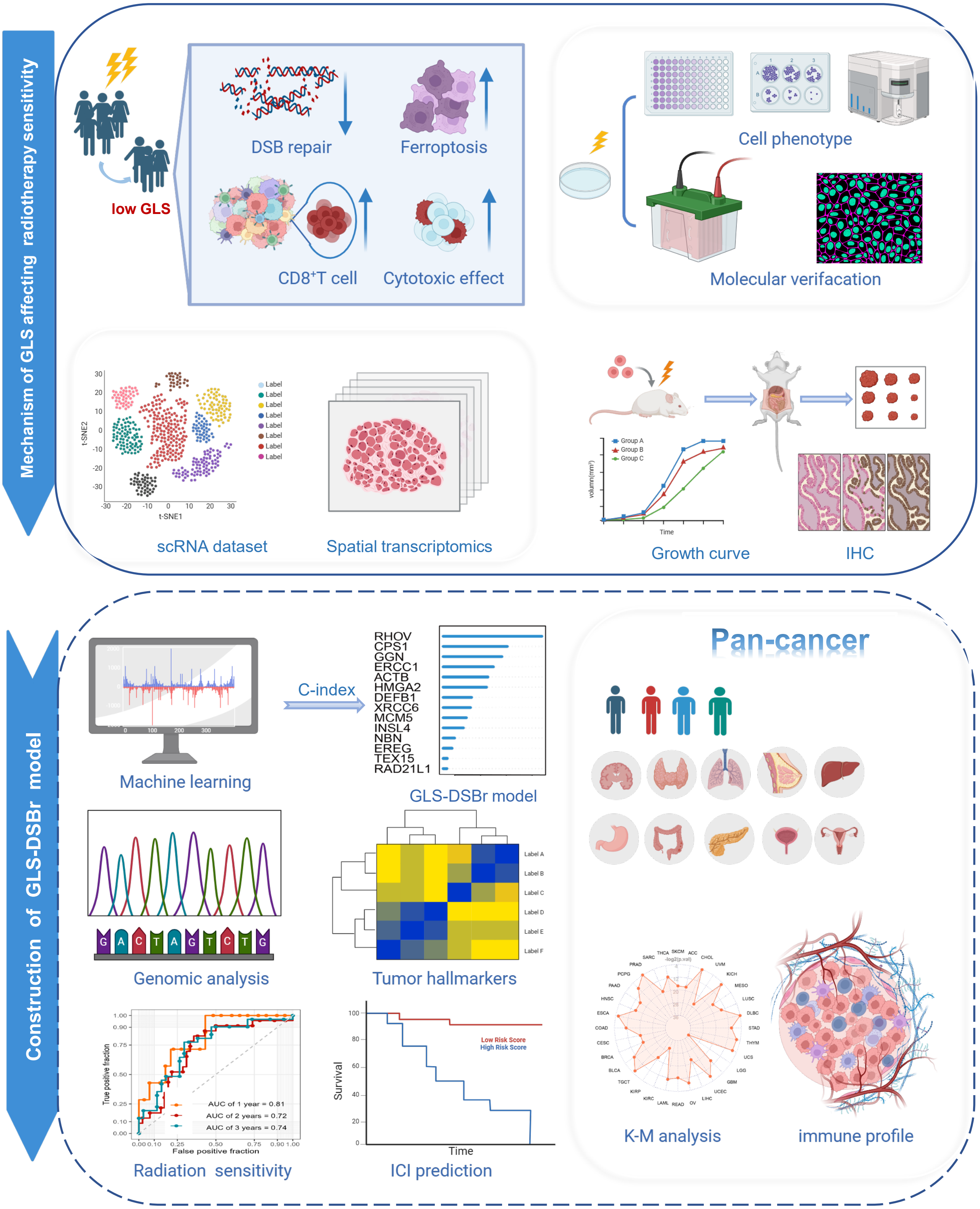

Figure S1. The graphical abstract for this research.

Figure S2. The relationship between glutamine metabolism and DSB repair in bulk datasets

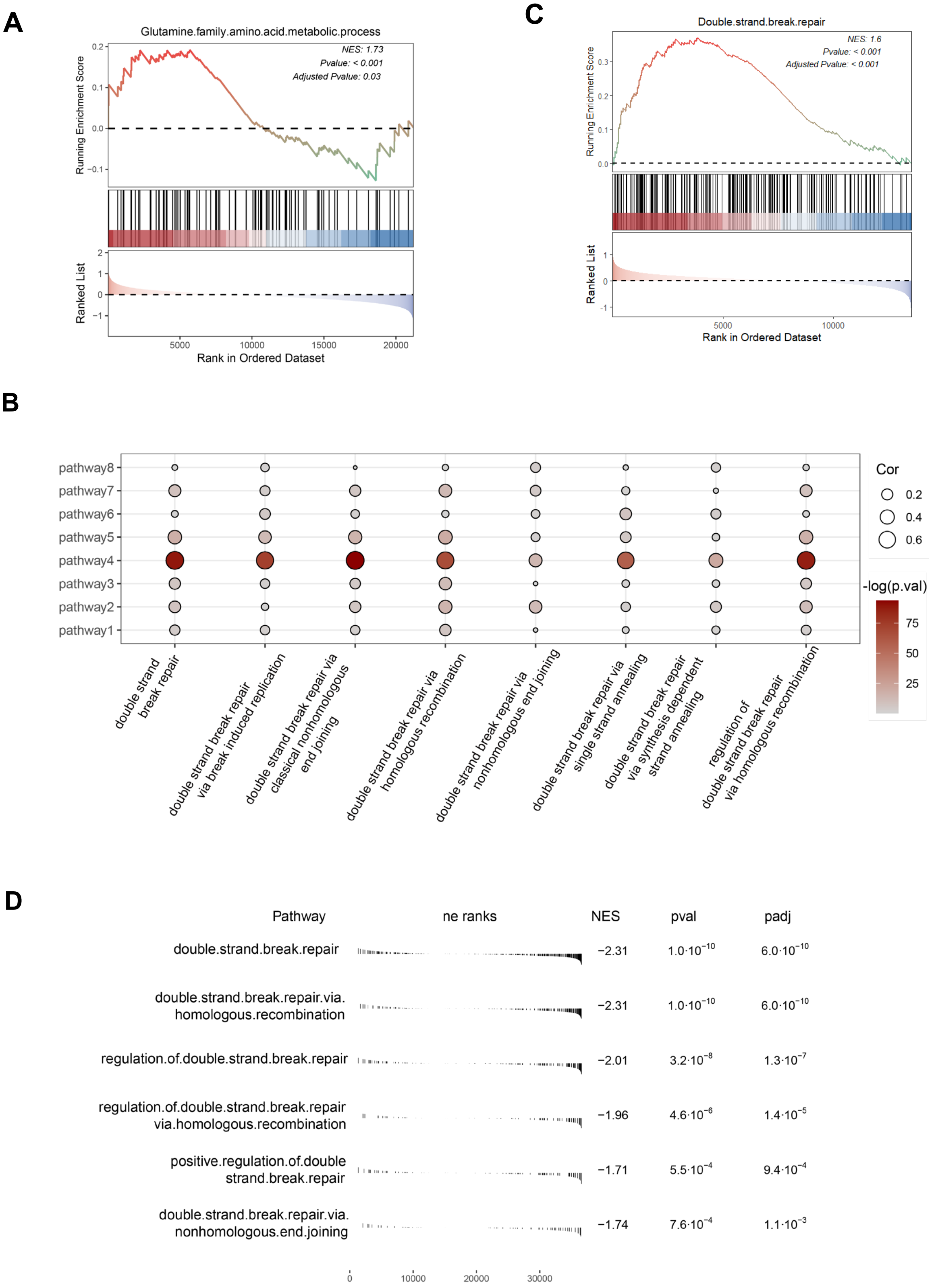

Figure S2. The relationship between glutamine metabolism and DSB repair in bulk datasets.

(A) GSEA results of the glutamine family amino acid metabolism in radioresistant and radiosensitive patients.

(B) The correlation of glutamine-metabolism related pathways and DSB repair related pathways in TCGA_LUAD.

(C) GSEA results of the DSB repair gene set in radiotherapy-treated patients with varying glutamine metabolism activity.

(D) GSEA results of the DSB repair related gene sets between varying glutamate biosynthetic flux in TCGA_LUAD.

Figure S3. The relationship between glutamine metabolism and TME

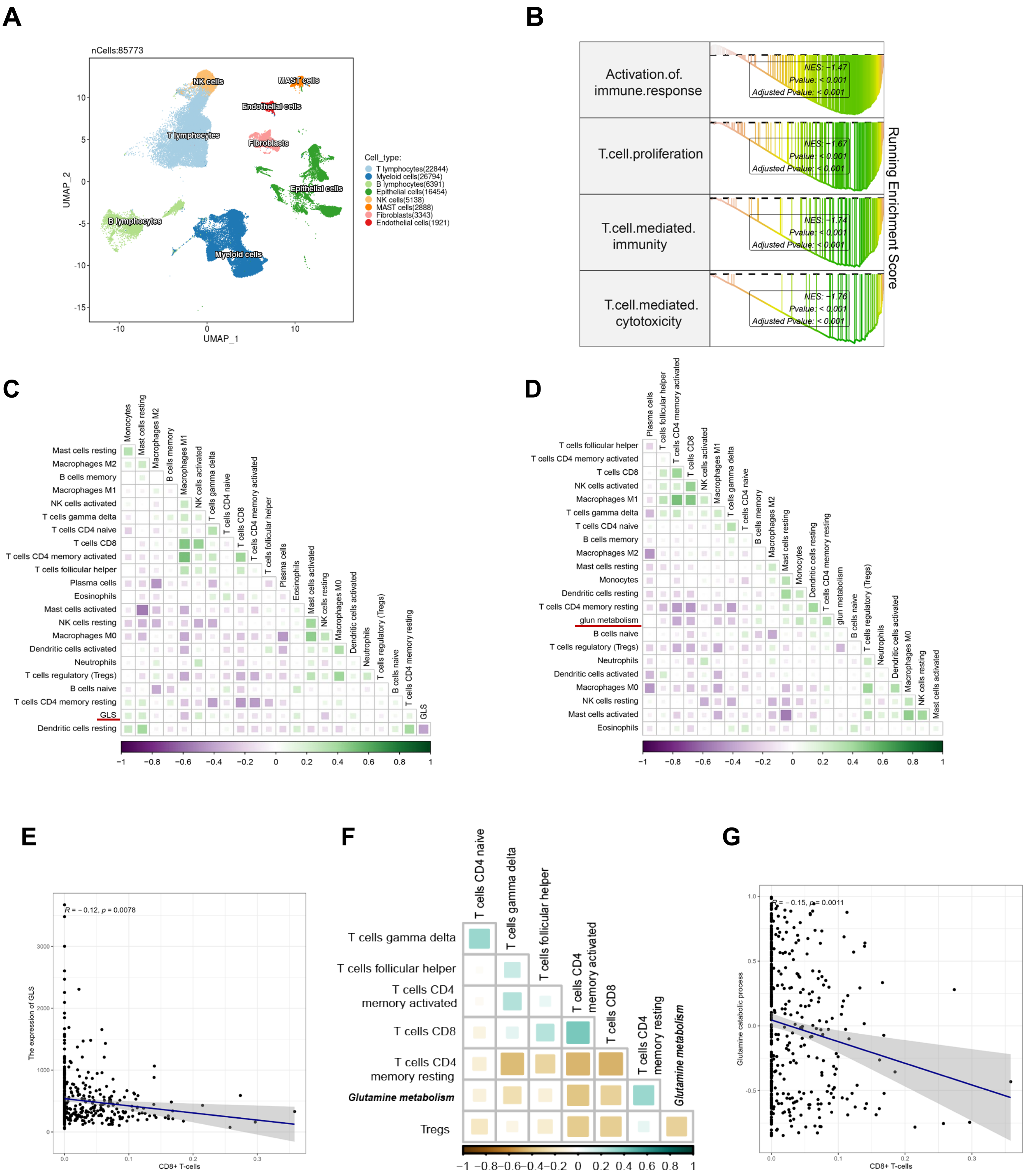

Figure S3. The relationship between glutamine metabolism and TME

(A) Single-cell profile of lung tissue.

(B) GSEA results of the immune-related gene sets in GLS-low and GLS-high patients in TCGA_LUAD.

(C) The correlation between GLS and immune cells.

(D) The correlation between glutamine-metabolism and immune cells.

(E) The correlation of GLS expression and $CD8^+$ T cells.

(F) The correlation between glutamine-metabolism and T cell subpopulations.

(G) The correlation of GLS glutamine-metabolism and $CD8^+$ T cells.

Figure S4. The impact of GLS expression in tumor cells on immune cell infiltration and glutamine metabolism in TME

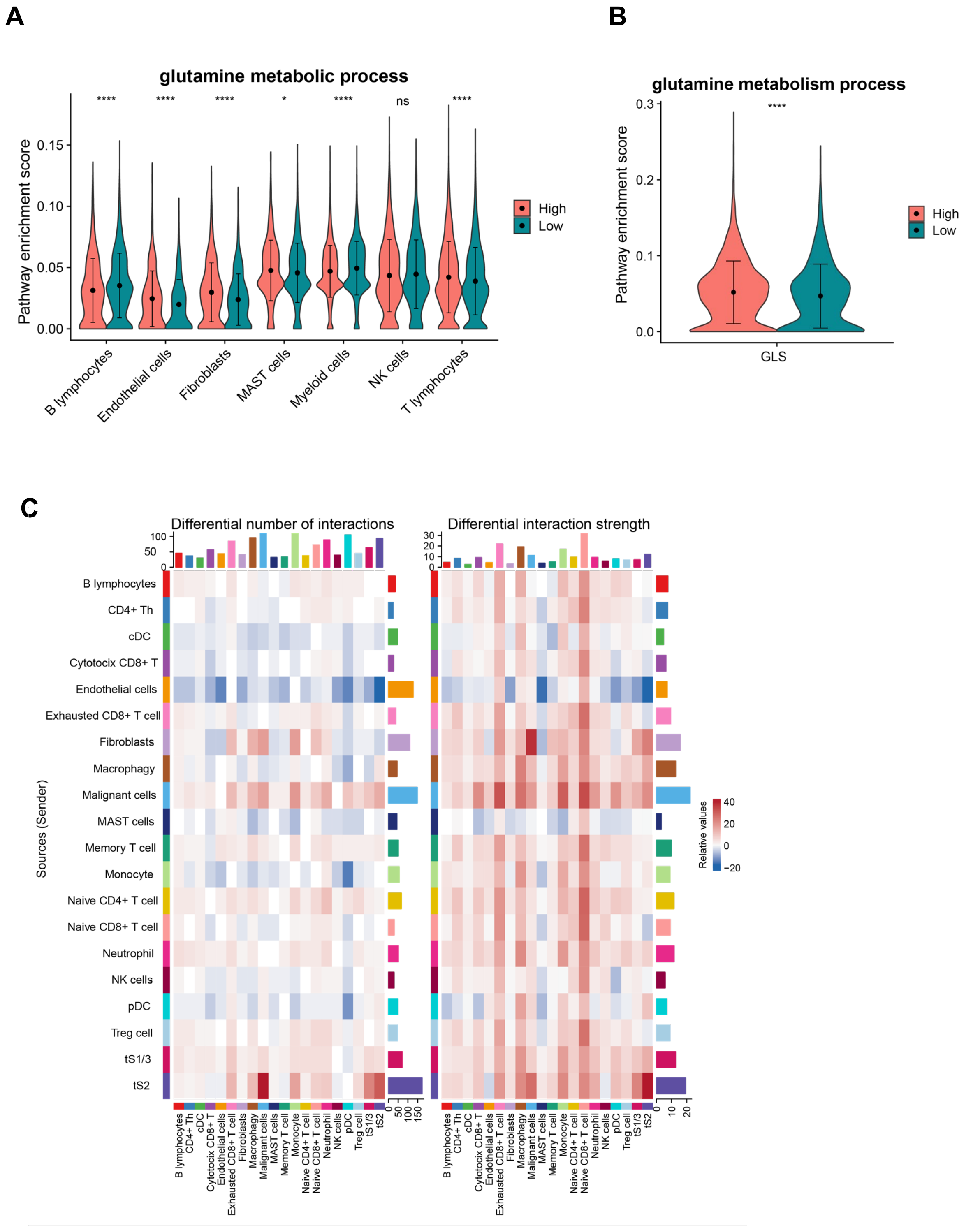

Figure S4. The impact of GLS expression in tumor cells on immune cell infiltration and glutamine metabolism in TME.

(A) Glutamine metabolic activity across immune cells between GLS-high and GLS-low groups.

(B) Glutamine metabolic activity across malignant cells between GLS-high and GLS-low groups.

(C) Details of the comparison of cell-to-cell interactions between the GLS-high and GLS-low groups.

Wilcox rank-sum test was used for panel (A), (B).

Figure S5. The impact of tumor cell GLS expression on immune cell infiltration in spatial transcriptomics

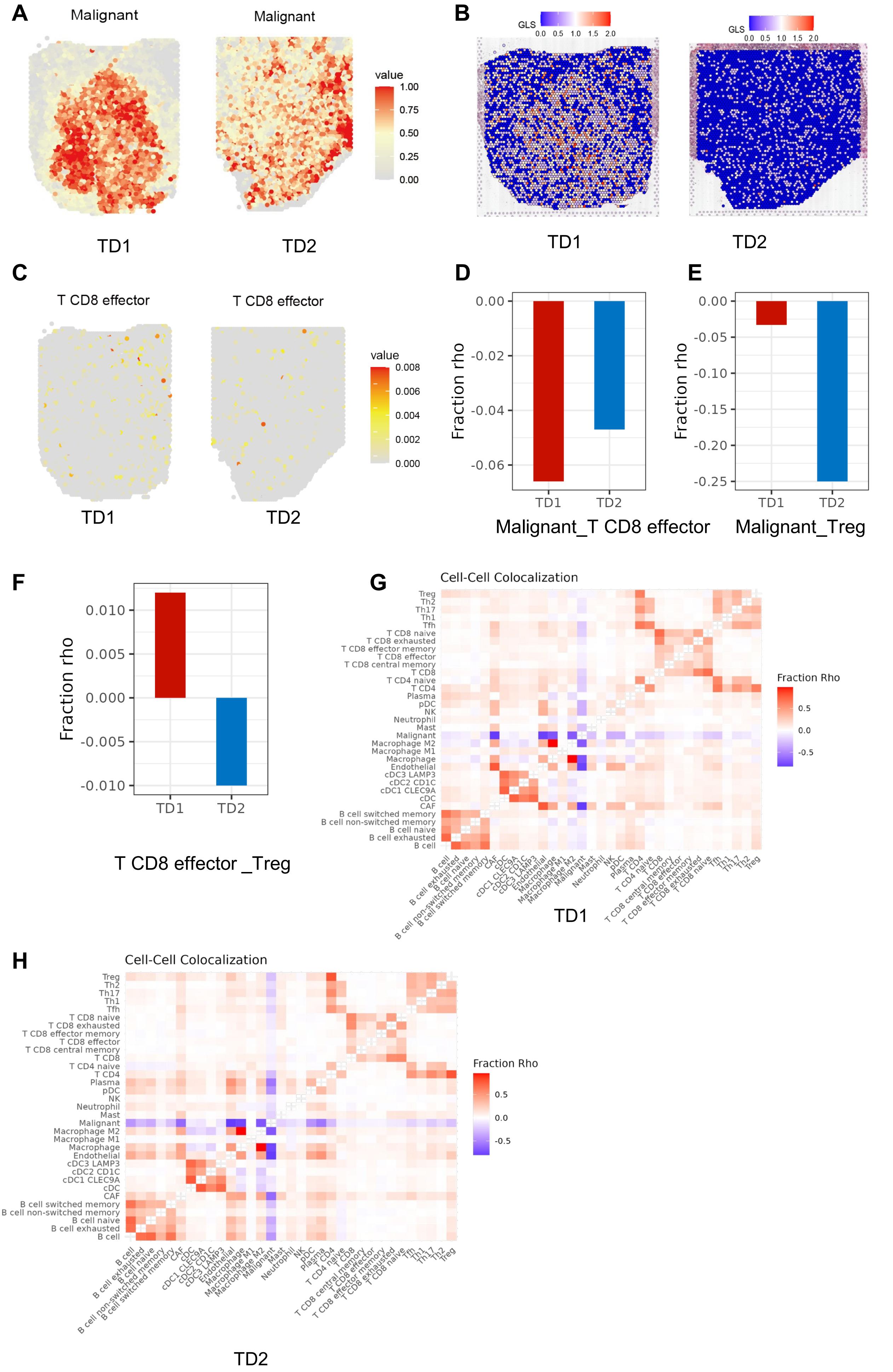

Figure S5. The impact of tumor cell GLS expression on immune cell infiltration in spatial transcriptomics.

(A) Spatial distribution of malignant cells.

(B) Spatial expression of GLS in TD1 and TD2 tumor tissues, separately.

(C) Infiltration of effector T cells in malignant tissue.

(D) Correlation coefficients of co-localization between malignant cells and effector T cells in TD1 and TD2.

(E) Correlation coefficients of co-localization between malignant cells and Tregs, in TD1 and TD2.

(F) Correlation coefficients of co-localization between effector T cells and Tregs in TD1 and TD2.

(G) The profile of co-localization coefficients of cell types in TD1.

(H) The profile of co-localization coefficients of cell types in TD2.

Figure S6. Inhibition of GLS increases ROS level in LUAD cells

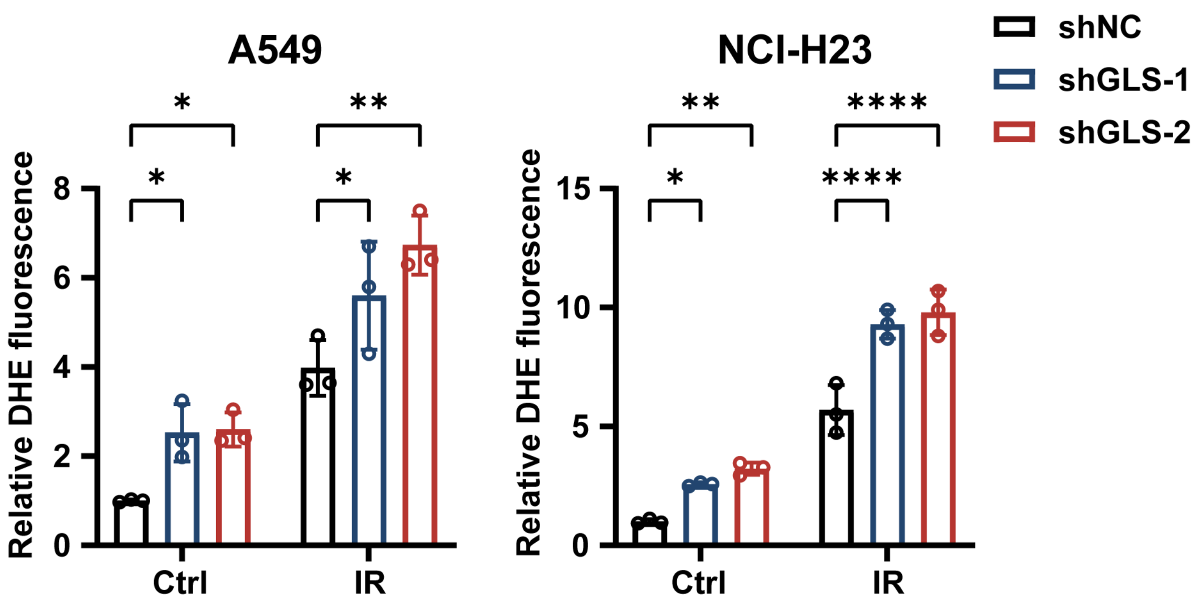

Figure S6. Inhibition of GLS increases ROS level in LUAD cells.

Intracellular ROS levels detected by DHE fluorescent probe in control and GLS-knockdown A549 and NCI-H23 cells with or without exposure to ionizing radiation (10 Gy).

All data are presented as mean ± S.D (n = 3). Two-way ANOVA test was used. IR, ionizing radiation; NC, negative control; DHE, dihydroethidium.

Figure S7. The construction and validation of GLS-DSBr model

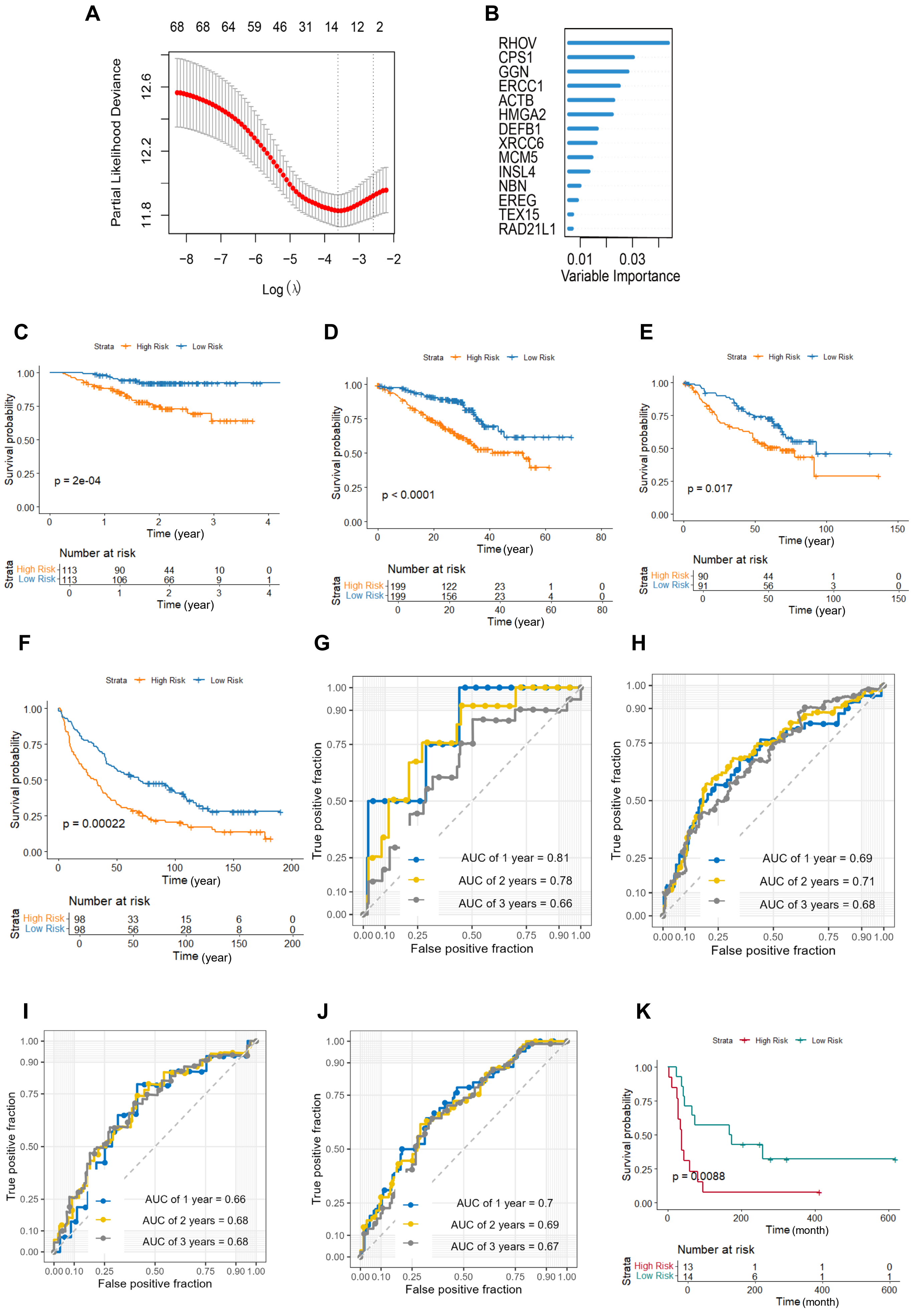

Figure S7. The construction and validation of GLS-DSBr model.

(A) The selection of optimal regularization parameter λ by lasso algorithm.

(B) The importance of mode genes.

(C-F) Kaplan-Meier survival plot based on GLS-DSBr score in GSE31210, GSE72094, GSE50081, GSE37745.

(G-J) The ROC curve of GLS-DSBr model in GSE31210, GSE72094, GSE50081, GSE37745.

(K) Kaplan-Meier survival plot based on GLS-DSBr score in patients with immunotherapy of GSE13522.

Figure S8. Genomic analysis and correlation analysis of GLS-DSBr model

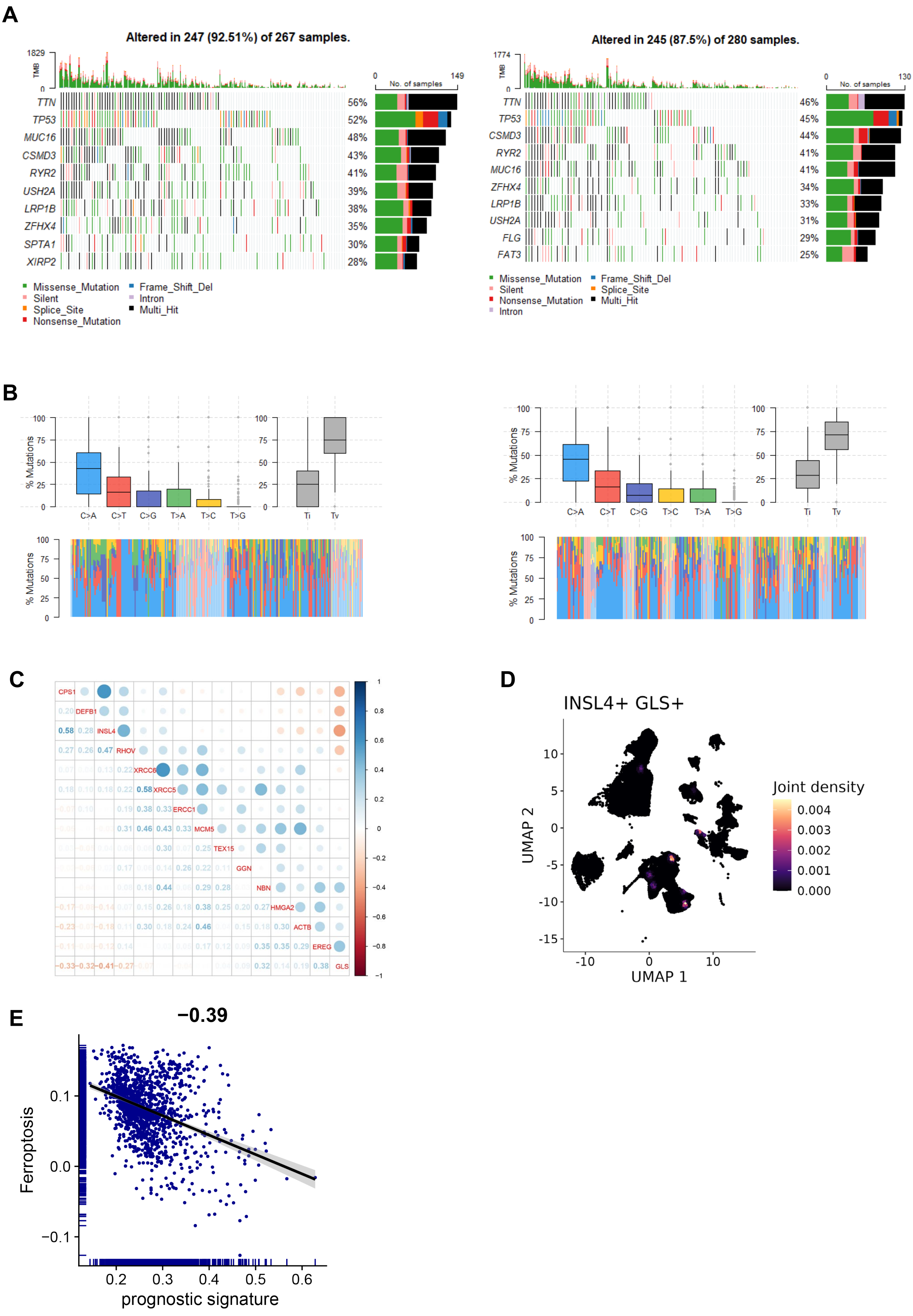

Figure S8. Genomic analysis and correlation analysis of GLS-DSBr model.

(A) The characteristics of CNV in high- and low-risk group, separately

(B) The situation of SNP types in 2 groups.

(C) Correlation between prognostic model genes and GLS expression.

(D) Co-expression of representative prognostic gene (INSL4) with GLS.

(E) Correlation between risk score and ferroptosis in tumor cells.

CNV, copy number variation; SNP, single nucleotide polymorphism.

Figure S9. Application of the GLS-DSBr model across pan-cancer

A

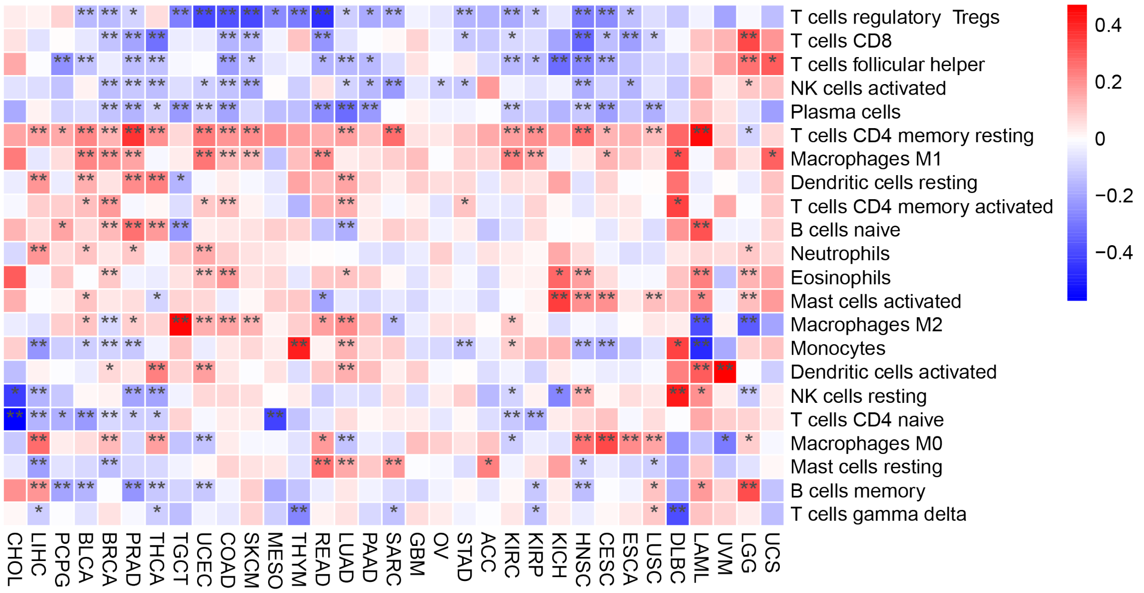

B

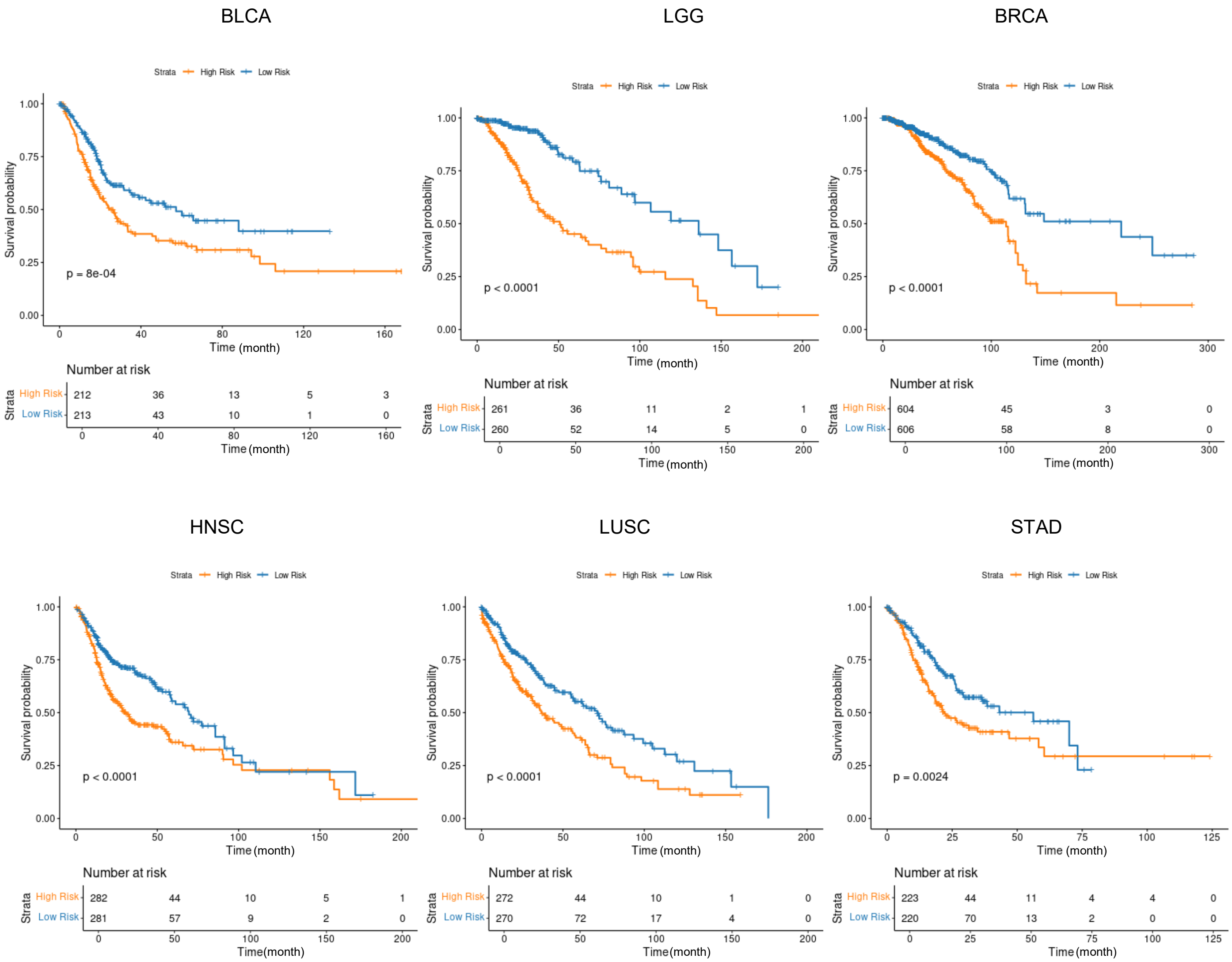

Figure S9. Application of the GLS-DSBr model across pan-cancer.

(A) Correlation between GLS expression and immune cell infiltration across pan-cancer.

(B) Kaplan-Meier survival plots for representative cancer types, including BLCA, LGG, BRCA, HNSC, LUSC and STAD.

BLCA, bladder urothelial carcinoma; LGG, brain lower grade glioma; BRCA, breast invasive carcinoma; HNSC, head and neck squamous cell carcinoma; LUSC, lung squamous cell carcinoma; STAD, stomach adenocarcinoma.

Figure S10. Relationship between the GLS-DSBr model and the TME across pan-cancer

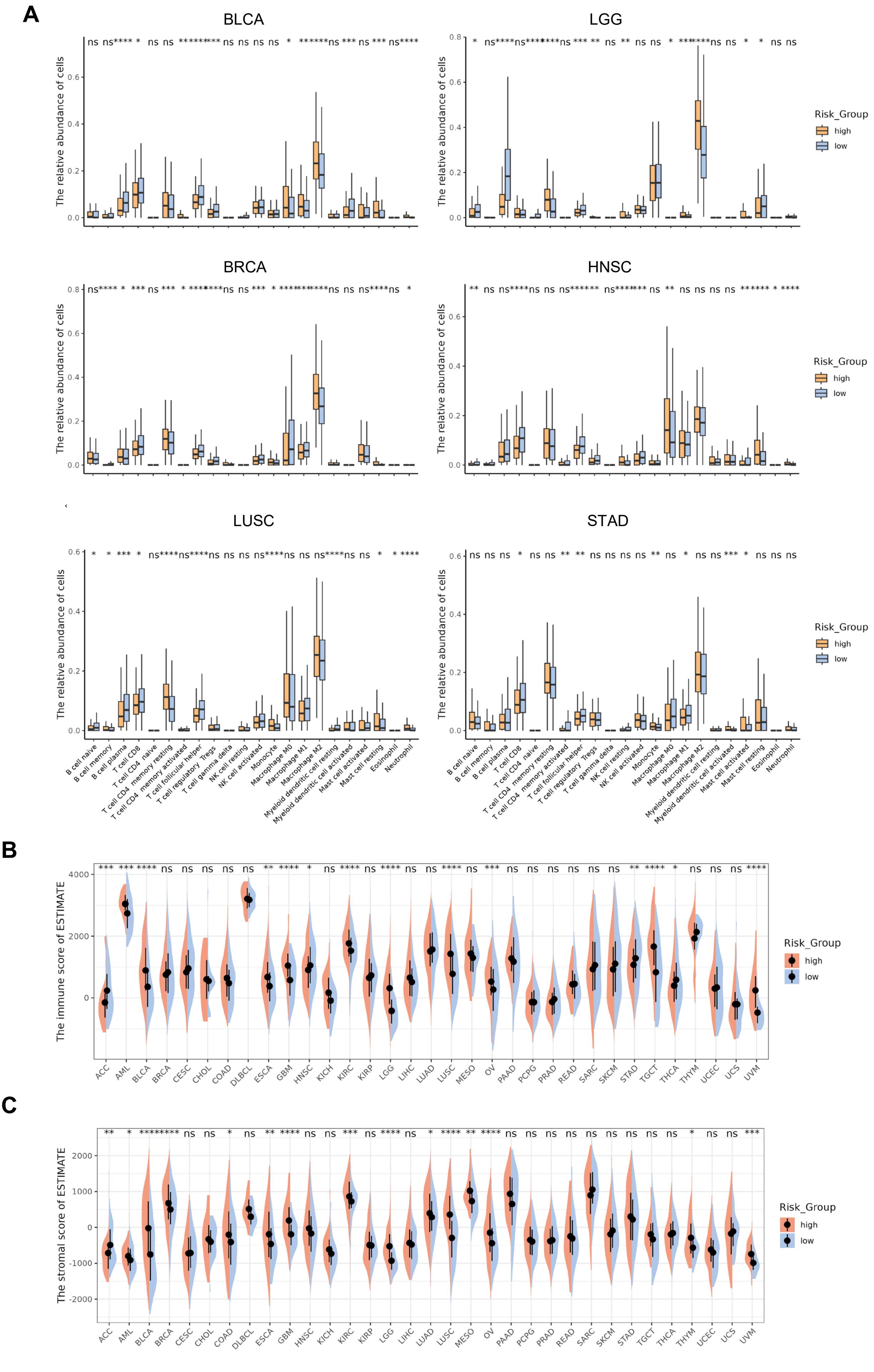

Figure S10. Relationship between the GLS-DSBr model and the TME across pan-cancer.

(A) Variations in infiltrated immune cell between groups at high and low risk for representative cancer types.

(B) Estimate immune scores across pan-cancer.

(C) Estimate stromal scores across pan-cancer.

Wilcox rank-sum test was used. BLCA, bladder urothelial carcinoma; LGG, brain lower grade glioma; BRCA, breast invasive carcinoma; HNSC, head and neck squamous cell carcinoma; LUSC, lung squamous cell carcinoma; STAD, stomach adenocarcinoma.
